# Super-Resolved Nuclear Magnetic Resonance Spectroscopy

**DOI:** 10.1038/s41598-017-09884-w

**Published:** 2017-08-29

**Authors:** Satish Mulleti, Amrinder Singh, Varsha P. Brahmkhatri, Kousik Chandra, Tahseen Raza, Sulakshana P. Mukherjee, Chandra Sekhar Seelamantula, Hanudatta S. Atreya

**Affiliations:** 1 Department of Electrical Engineering, Indian Institute of Science, Bangalore, 560012 India; 2 NMR Research Centre, Indian Institute of Science, Bangalore, 560012 India; 3 Department of Biosciences, Indian Institute of Technology, Roorkee, 247667 India

## Abstract

We present a novel method that breaks the resolution barrier in nuclear magnetic resonance (NMR) spectroscopy, allowing one to accurately estimate the chemical shift values of highly overlapping or broadened peaks. This problem is routinely encountered in NMR when peaks have large linewidths due to rapidly decaying signals, hindering its application. We address this problem based on the notion of finite-rate-of-innovation (FRI) sampling, which is based on the premise that signals such as the NMR signal, can be accurately reconstructed using fewer measurements than that required by existing approaches. The FRI approach leads to super-resolution, beyond the limits of contemporary NMR techniques. Using this method, we could measure for the first time small changes in chemical shifts during the formation of a Gold nanorod-protein complex, facilitating the quantification of the strength of such interactions. The method thus opens up new possibilities for the application and acceleration of multidimensional NMR spectroscopy across a wide range of systems.

## Introduction

Nuclear magnetic resonance (NMR) spectroscopy is a powerful technique for structural and dynamic studies of molecules. An important step in the analysis of NMR spectroscopic data is accurate estimation of the chemical shift values (or frequencies) of peaks observed in the spectrum. The solution is straightforward when the peaks do not overlap and when the signal-to-noise ratio (S/N) is high. However, it is a challenging task when peaks overlap significantly, with low S/N further compounding the issue. This problem is frequently encountered in systems involving large proteins and their complexes^1^, materials in solid state^2^, and in metabolomics^3^ where the sample contains a mixture of a large number of compounds with varying S/N. The resolution in NMR is directly proportional to the duration for which the signal is acquired and is considered to be limited by the intrinsic linewidth of the peaks^4^. For achieving good resolution, the NMR signal or the free induction decay (FID) is acquired typically with a large number of sampling points and processed using apodization, linear prediction or line-shape fitting^5–7^. However, these methods become unsuccessful if the signal decays rapidly due to transverse relaxation resulting in large linewidths. Further, acquiring spectra with a good S/N and with a large number of sampled points in FID entails increased measurement time. While approaches such as transverse relaxation optimized spectroscopy (TROSY)^8^ have been established for improving spectral resolution in large molecular weight systems in solution and high speed magic angle spinning for solid state NMR^9^, resolving broad or overlapping peaks remains a bottleneck for application of NMR.

During the past decade, efficient methods have been developed to sample and reconstruct structured signals that have a parsimonious representation in a certain bases^10–13^ or possess a finite number of degrees of freedom^14, 15^ over a given interval. The latter type of signals are called finite-rate-of-innovation (FRI) signals^14^. FRI-based reconstruction approaches have been used in several applications, such as ultrasound imaging^16, 17^, radio astronomy^18^, radar imaging^19, 20^, light detection and ranging^21^, frequency-domain optical-coherence tomography (FDOCT)^22–24^, source localization^25, 26^, compression of electrocardiogram signals^27^, and curve fitting^28, 29^, where it has been shown that the FRI structure results in super-resolved reconstruction starting from a smaller number of measurements in comparison with the classical Fourier-based approaches.

In this paper, we demonstrate that NMR signals (i.e., FIDs) have the FRI property and propose a new autocorrelation-based spectral estimation technique to accurately estimate the chemical shifts in the presence of exponential damping. We show that one requires much less number of uniform measurements of the FID to accurately estimate its parameters and achieve super-resolved NMR spectra, surpassing the limits of contemporary approaches. We refer to this approach as ‘*FRI-NMR*’. The ability to resolve broad or overlapping peaks is accompanied by a reduction in the number of measurements, thereby bringing down the measurement time, which is crucial for higher dimensional NMR analysis.

## Results

### Comparison of FRI-NMR with linear prediction and the Fourier transform

The principle of FRI-NMR is explained in detail in Section S1 of Supporting Information. The method uses the structure of the FID (exponentially damped sinusoids) as prior knowledge together with a new autocorrelation-based parameter estimation technique^30, 31^. Modelling the noise in the FID as additive white Gaussian, effectively, the noise contribution in the autocorrelation gets restricted to the zero-lag, while retaining its sum-of-exponential property (described in Section S1). This property is used to resolve closely spaced frequencies with high accuracy, which is otherwise not possible using the standard Fourier or linear prediction methods.

**Figure 1:**
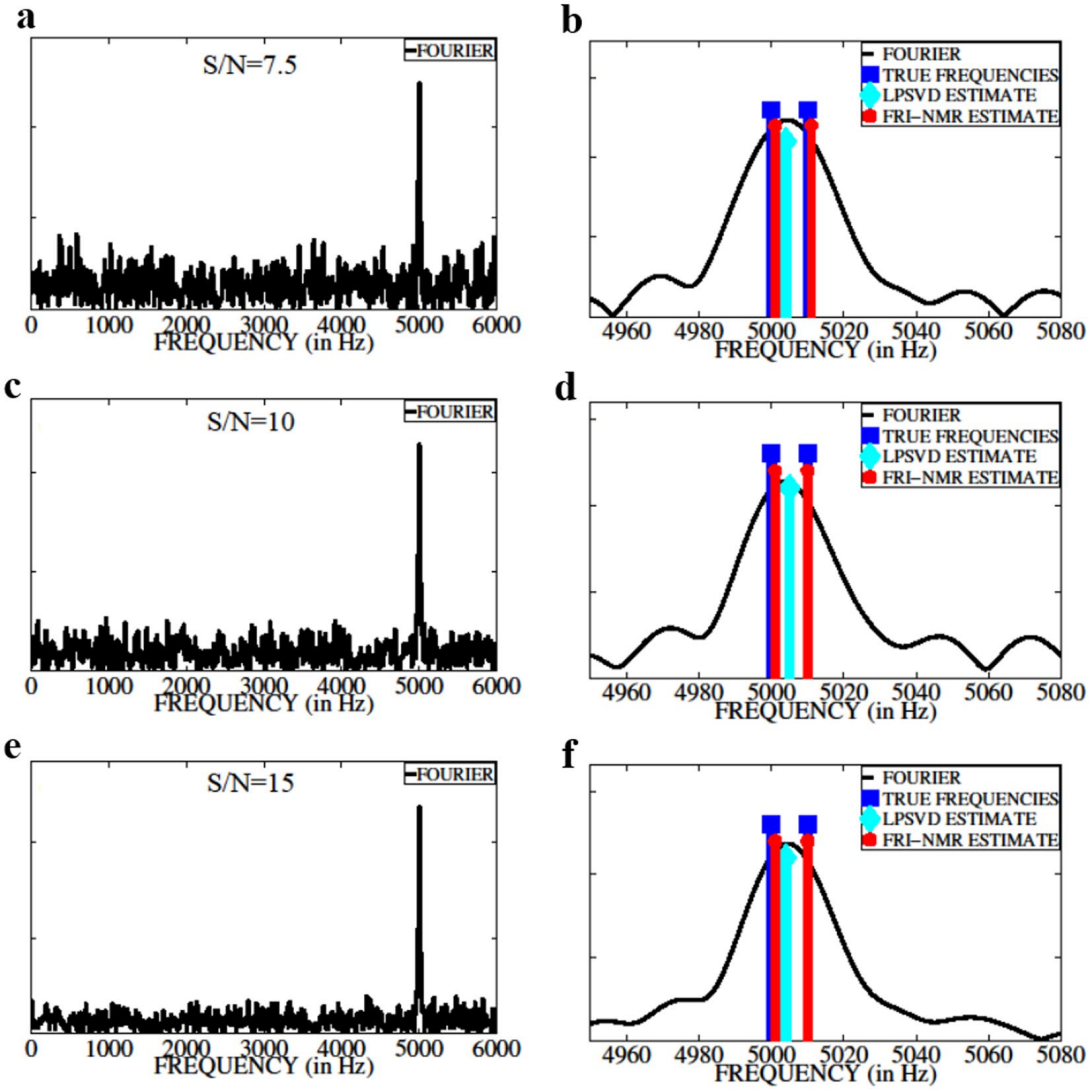
A comparison of frequency-resolution capability of different methods. (**a**), (**c**), and (**e**) show the Fourier-transformed magnitude spectra of 600 noisy FID samples consisting of two frequencies separated by Δ*F* = 10 Hz and sampled at 12 kHz for S/N = 7.5; 10; 15, respectively. Figs (**b**), (**d**), and (**f**) show the corresponding zoomed-in plots together with the true frequencies (in blue), FRI-NMR estimates (in red), and LPSVD estimates (in cyan). FRI-NMR is able to resolve frequencies that are below the resolution limit (20 Hz) of the Fourier and Linear prediction method for different S/N levels.

The resolution capability of FRI-NMR can be gleaned from its comparison with the often used standard Fourier and linear prediction methods (LPSVD). We consider a FID of the form:^4^

$$\tilde{f}(n{T}_{s})=f(n{T}_{s})+w(n),$$

$$\qquad =\sum _{l=1}^{L}{a}_{l}{e}^{(-{\alpha }_{l}+j{\omega }_{l})n{T}_{s}}+w(n),$$

where *ω*_*l*_ are the *L* frequencies (chemical shifts) that are present in the FID, *α*_*l*_ are the relaxation rates (1/T_2_), *a*_*l*_ are the respective amplitudes, and *T*_*s*_ is the dwell time (inverse of the sampling frequency). Consider two frequencies with *a*_1_ = *a*_2_ = 1, *α*_1_ = *α*_2_ = 1. The frequencies of the two components are chosen as *F*_1_ = 5000 Hz and *F*_2_ = 5000 + Δ*F* Hz, where Δ*F* is the frequency separation. The FID is sampled at 12 kHz and a total of *N* = 600 complex points are collected. The FID is corrupted by zero-mean additive white Gaussian noise (AWGN). The noise variance ${\sigma }_{w}^{2}$ is chosen in order to achieve a given S/N. We have chosen similar amplitudes and damping factors for the two FID components so that they have equal S/N. The performance of FRI-NMR in resolving the two frequencies is assessed by varying Δ*F* below the Fourier resolution limit given by $\frac{1}{{T}_{obs}}\le \frac{1}{(N-1){T}_{s}} \sim \,20$ Hz. Figure 1 shows the spectra for ΔF = 10 Hz and S/N = 7.5, 10, 15. The right panel shows the magnified spectra with the true frequencies (in blue), FRI-NMR estimates (in red), and LPSVD estimates (in cyan). The spectra corresponding to linear prediction (LPSVD) and FRI-NMR frequency estimates are plotted by placing Dirac impulses of known amplitudes at the estimated frequencies. We observe that at all three S/N levels, Fourier transform and linear prediction methods are incapable of resolving the two frequencies, which are closer than the resolution limit governed by the length of the FID. On the other hand, the FRI-NMR approach is able to resolve the two frequencies. Indeed, for shorter FIDs and in presence of high noise, the Fourier transform peak shifts away from the correct frequency, which is illustrated in Fig. S1 of Supporting Information. A detailed comparison of the different methods is presented in Section S2 of Supporting Information.

**Figure 2:**
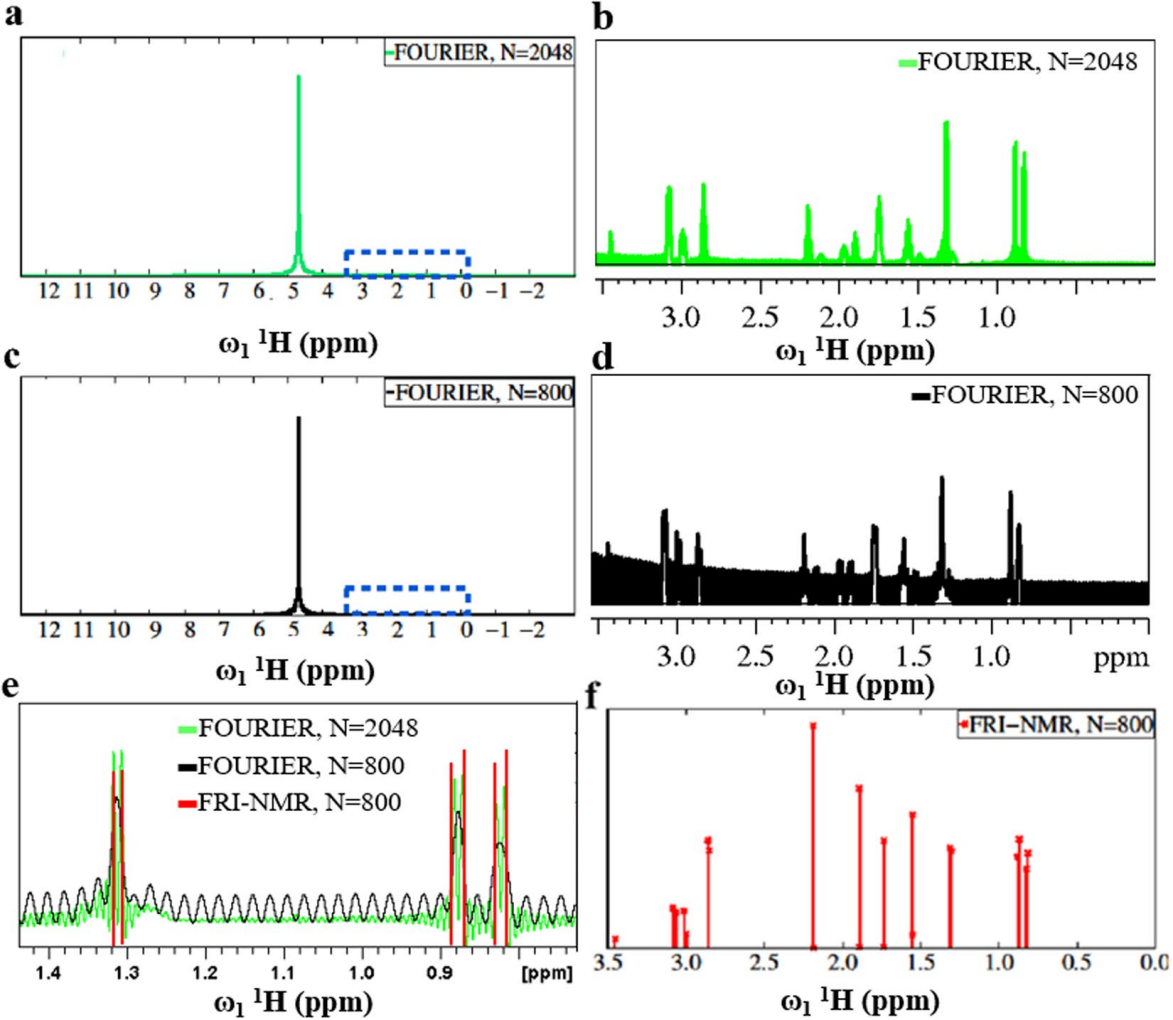
Comparison of the Fourier-transformed spectrum and FRI-NMR in the estimation of frequencies in a ^1^H NMR spectrum. (**a**) High-resolution Fourier-transformed spectrum using 2048 complex points of the FID; and (**b**) a magnified region highlighted by the blue rectangle in (**a**); (**c**) and (**d**) show the corresponding low-resolution Fourier spectrum obtained using 800 complex points and its zoomed-in region highlighted by the blue rectangle, respectively (similar to (**a**) and (**b**)); (**e**) a zoomed-in region of the spectrum showing the super-resolution capability of FRI-NMR. The Dirac impulses (stem plot shown in red) indicate the FRI-NMR chemical shift estimates corresponding to the regions shown in (**b**) and (**d**). The 1D ^**1**^H NMR spectrum of a mixture of amino acids was acquired with 16,384 points and 2 transients with relaxation delay of 2 seconds between scans.

The super-resolution capability of the FRI-NMR approach was further assessed on a sample containing a mixture of the following amino acids in 95% H_**2**_O at a concentration of 0.5 mM each: Alanine, Valine, Arginine, Lysine, Histidine and Glutamic acid. Figure 2a shows the high-resolution Fourier transformed 1D ^**1**^H NMR spectrum computed using 2048 complex points in the FID and Fig. 2b shows a magnified portion (highlighted in Fig. 2a). The figures show that in the Fourier transformed 1D ^**1**^H spectrum the signals are masked/distorted by the large ^**1**^H signal of H_2_O, which makes it difficult to accurately estimate the chemical shifts of the signals from the compounds.

Next, the first 800 samples of the FID were selected, which corresponds to about 40% of the total number of points and Fourier transformed to yield an average S/N of 10 for the non-water ^1^H signals. The resulting low S/N and resolution is noticeable in Fig. 2c and 2d, the latter showing an expanded version of the spectrum. In such a scenario, the FRI-NMR approach is able to correctly extract the chemical shift values as shown in Fig. 2e. Notably, the six peaks shown in Fig. 2e, two each at around 0.81 ppm, 0.88 ppm and 1.3 ppm, which are super-resolved by the FRI-NMR method, are either reduced to a single peak or have high overlap in the resolution-limited Fourier spectrum. The chemical shifts are accurately estimated using the FRI-NMR approach, in addition to correctly maintaining the amplitudes on a relative scale (Fig. 2e). This experiment illustrates the robustness of the proposed FRI-NMR approach.

### FRI-NMR estimation of frequencies in two-dimensional NMR spectrum

FRI-NMR is ideally suited for multidimensional NMR spectral analysis, where the linewidth and resolution in the indirect dimension depend on transverse relaxation and/or number of points sampled due to measurement time constraints^4^. This is illustrated using 2D heteronuclear single quantum correlation (HSQC) NMR spectrum of two proteins, Ubiquitin (8.6 kDa) and N-terminal domain of p50 (39–245; 23 kDa) (p50-NTD)^32^. The 2D HSQC spectrum or its variant, the 2D heteronuclear multiple quantum coherence (HMQC), is routinely acquired for resonance assignment of proteins and serves as an important spectrum for monitoring structural and dynamic processes^4^.

**Figure 3:**
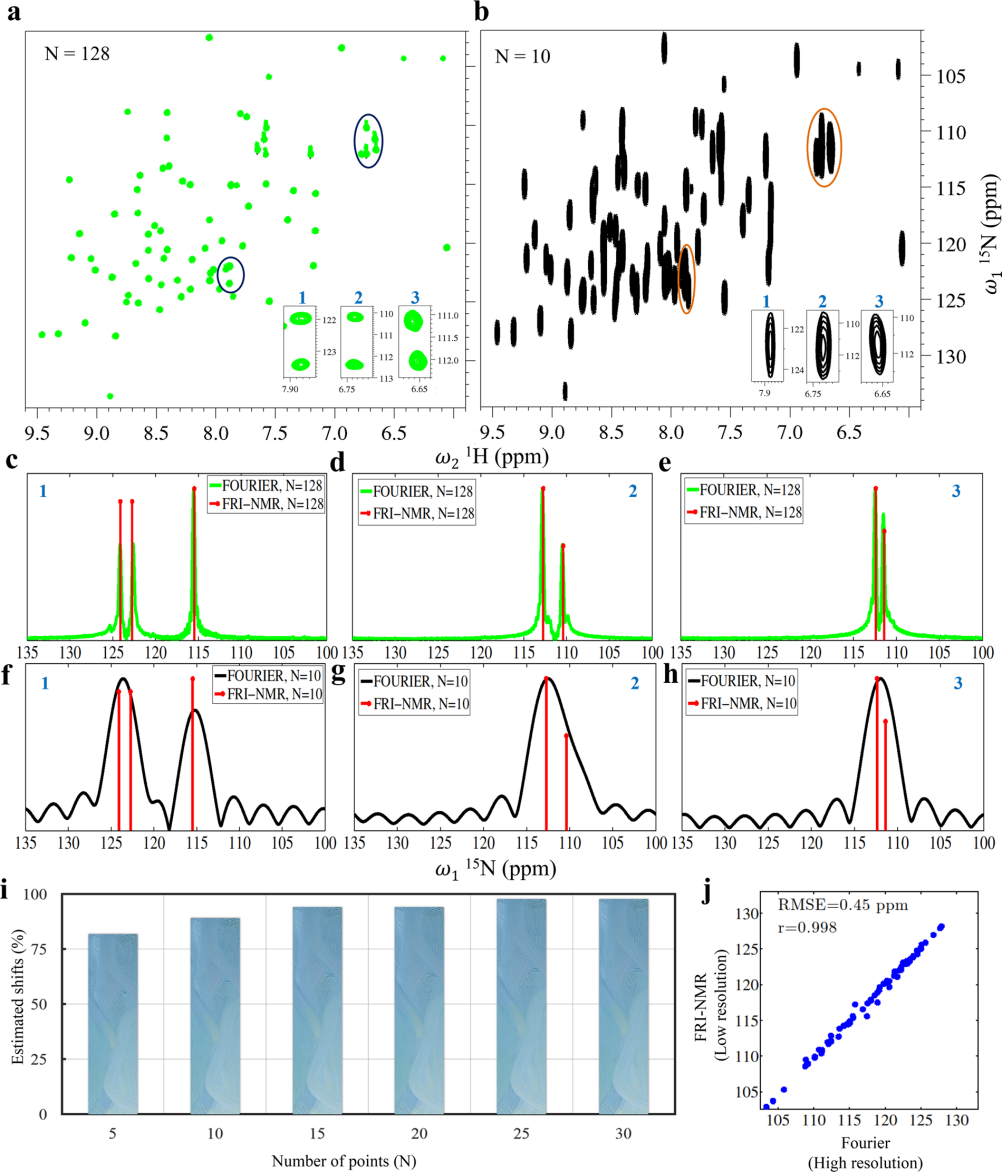
Resolving capability of FRI-NMR for proteins. (**a**) The 2D [^15^N-^1^H] HSQC spectrum of Ubiquitin acquired with 128 complex points along the indirect dimension (indicated as *N* = 128). (**b**) A low-resolution spectrum obtained from (**a**) by considering the first 10 points in the FID along the ^15^N dimension (1). To illustrate the resolving capability of FRI-NMR, three regions with peaks that are not resolved in (**b**) (shown magnified at bottom-right) were chosen. For each of these three regions, the underlying overlapping frequencies along ω_1_ were estimated using FRI-NMR as shown in (**c–h**). In (**c–e**) the FRI-NMR estimates (red lines) are shown superimposed on the high-resolution 1D traces obtained from (**a**). In (**f–h**) the same frequencies are estimated by FRI-NMR from FID containing 10 points and are shown superimposed on the corresponding Fourier-transformed spectrum. (**i**) The percentage of peaks in Ubiquitin that was estimated by FRI-NMR using the first *N* points in the FID (*N* =  5 to 30). (**j**) The high correlation obtained between the chemical shift values estimated by FRI-NMR from the 10-point FID and the high-resolution 128-point FID. The corresponding RMSE value between the two sets is also indicated.

In the case of Ubiquitin, a high-resolution spectrum acquired with 128 complex points (Fig. 3a) was taken and processed with 10 complex points to mimic reduction in resolution by more than an order of magnitude due to line broadening from 20 Hz to 250 Hz (Fig. 3b). In order to estimate frequencies along the indirect (*F*_1_) dimension in the low-resolution spectrum using FRI-NMR, the 2D time-domain data was first Fourier transformed along the direct dimension (*F*_2_). Following this, for each peak in *F*_2_(*ω*_2_), the time-domain signal along *F*_1_(*ω*_1_) was chosen for frequency estimation. Comparing the two spectra shown in Fig. 3a and b, it is clear that due to the low resolution, the 2D spectrum obtained from 10 complex points along *F*_1 _cannot resolve a large number of peaks. However, FRI-NMR is able to accurately estimate both the position and amplitude of the peaks along the *F*_1 _dimension as depicted for three cases (shown in the inset in Fig. 3a and b and in Fig. 3c–h). Even if one were to consider doubling the length of the FID using linear prediction (which is usually done in multidimensional NMR^4^) or spectral reconstruction using non-uniform sampling, the peaks would not be resolved (shown in Figs S6 and S7). FRI-NMR allows us to go beyond the limitation imposed by the length of the FID for resolving chemical shifts.

Even with further reduction to just 5 complex points (corresponding to linewidths of ~500 Hz), about 82% of the chemical shifts could be estimated that matched the values obtained from the high-resolution spectrum. Due to reduced S/N, about 18% of the frequencies (out of the expected 72 peaks) could not be estimated. However, the number of estimated chemical shifts increased sharply as the number of points was increased. This is depicted in Fig. 3i in the form of a histogram plot.

In order to assess the accuracy of chemical shifts estimated by FRI-NMR, we compared the values of shifts obtained from low-resolution spectrum of Ubiquitin as described above (*N* = 10) with those obtained from the high-resolution spectrum (*N* = 128) (Fig. 3a,b). The comparison is shown in Fig. 3j, which indicates high correlation and low root mean-square error (RMSE) between the chemical shifts obtained from the two spectra. The chemical shifts estimated by FRI-NMR have an RMSE of 0.45 ppm (36 Hz) when the actual linewidths, which governs the Fourier limit of peak separation, are ~250 Hz.

**Figure 4:**
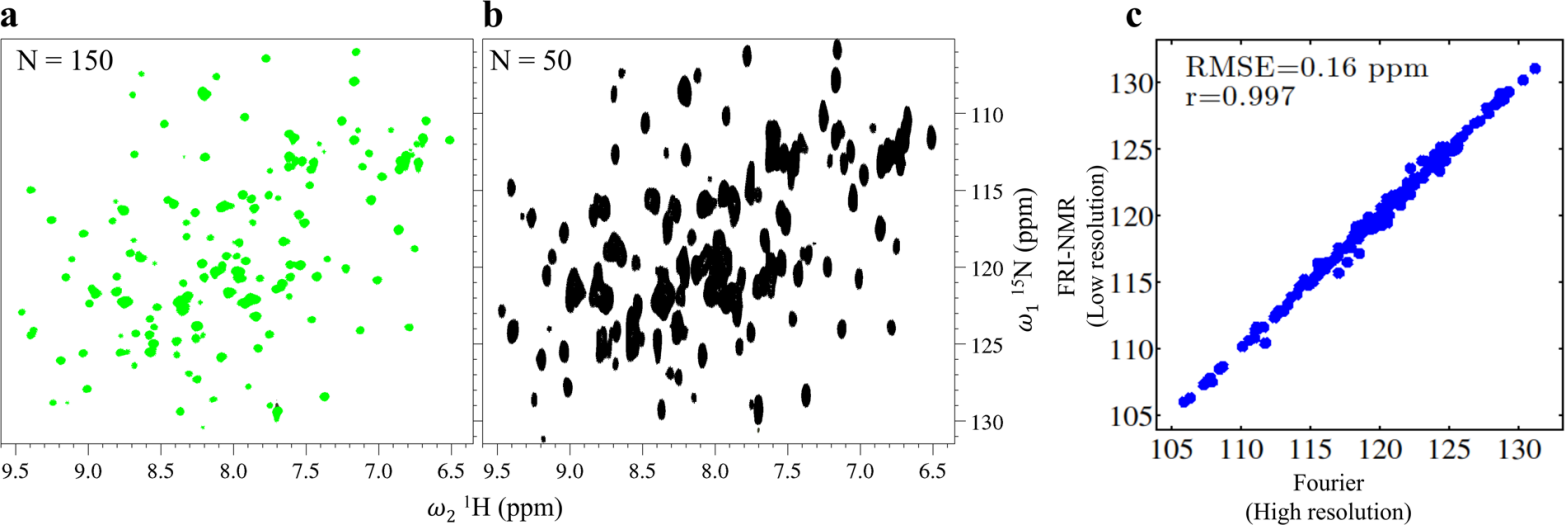
Accuracy of FRI-NMR for large proteins. (**a**) The 2D [^15^N-^1^H] HSQC spectrum of p50-NTD acquired with 150 complex points along the indirect dimension (indicated as *N* = 150). (**b**) A low-resolution spectrum obtained from (**a**) by considering the first 50 points in the FID along the ^15^N dimension (1). (**c**) The correlation obtained between the chemical shift value estimated by FRI-NMR from the 50-point FID and the high-resolution 150-point FID. The corresponding RMSE value between the two data sets is also indicated.

The FRI-NMR is useful for large proteins where the FID decays rapidly. It allows one to improve the S/N by increasing the number of scans for signal averaging while reducing the number of points sampled in the FID. This is exemplified for the protein p50-NTD (23 kDa). The 2D HSQC spectrum of the protonated form of p50-NTD is shown in Fig. 4a. The spectrum was acquired for 150 complex points in the indirect (^15^N) dimension resulting in a linewidth of ~20 Hz. However, with FRI-NMR using 50 complex points corresponding to larger linewidths of ~60 Hz, all the observed 175 chemical shifts could be estimated as accurately as the Fourier based estimation with an RMSE of 0.16 ppm (~13 Hz) (Fig. 4b). The estimation of number of frequencies is described in Section S3 of Supporting Information.

### Using FRI-NMR to quantify protein-gold nanorod interactions

**Figure 5:**
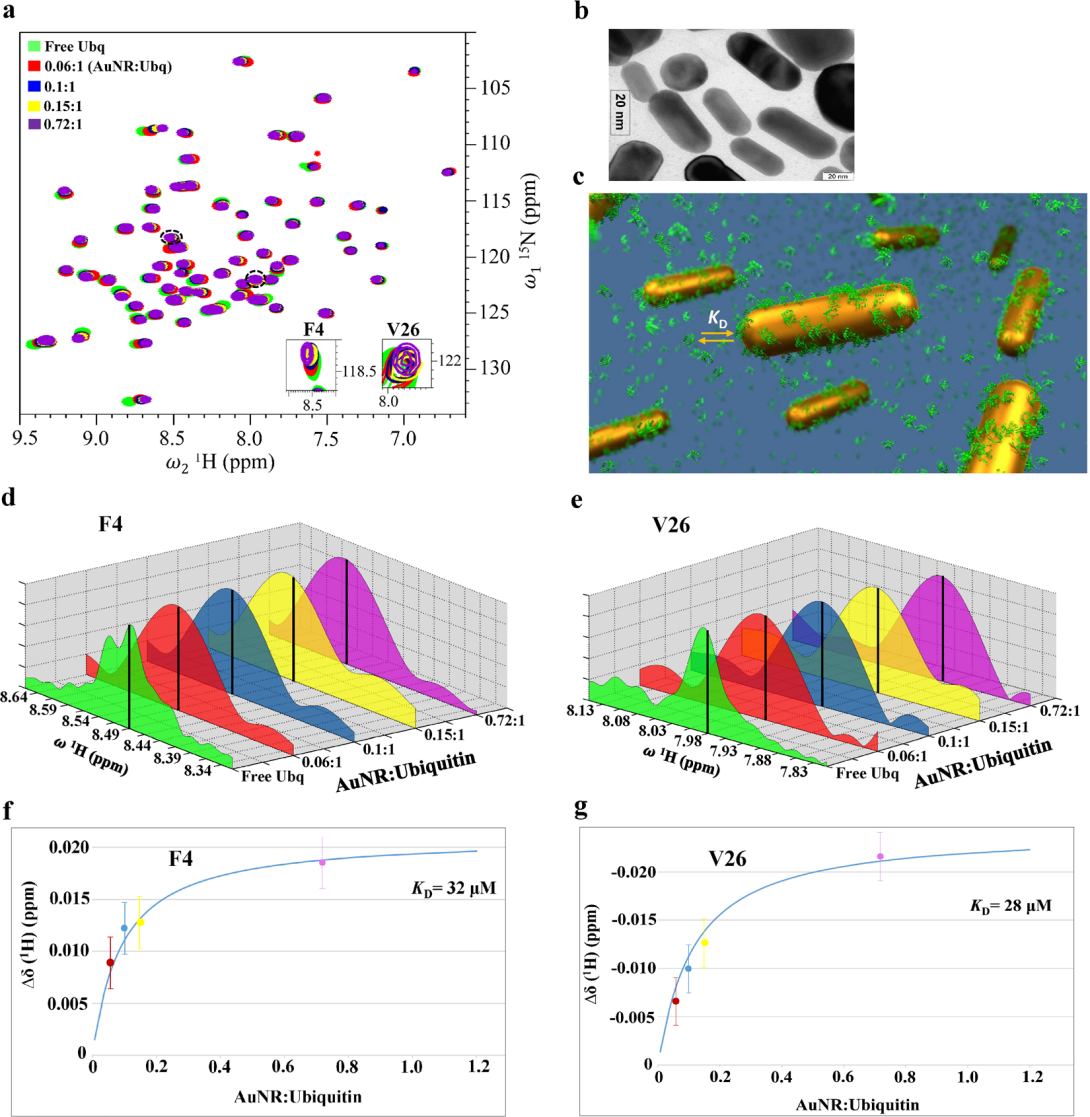
Application of FRI-NMR to study Ubiquitin-Gold nanorod interactions. (**a**) Overlay of the 2D [^15^N-^1^H] HSQC spectra of Ubiquitin acquired at different gold nanorod (AuNR) to protein concentration ratios; (**b**) A transmission electron microscopy (TEM) image indicating the dimensions of nanorods; (**c**) A schematic illustration of the protein-gold nanorod interaction depicting the adsorption of the protein on the nanorod surface and the exchange of the adsorbed protein molecules with those in the bulk with a dissociation constant (*K*_D_); (**d**) and (**e**) are the 1D traces along ^**1**^H (ω_2_) for the two residues (F4 and V26) shown magnified in (**a**) at different protein additions. The black vertical lines indicate the FRI-NMR estimates of chemical shifts. The high-resolution spectrum of free Ubiquitin is shown; (**f**), (**g**) are the corresponding plots of the change in the ^**1**^H chemical shift with respect to free Ubiquitin for different additions of the protein estimated using the FRI-NMR method. The continuous blue line corresponds to the fit of the equation used for determining the indicated *K*_D_ values (see Section S4 of Supporting Information).

The frequency estimation accuracy offered by FRI-NMR enabled the measurement of small changes in the chemical shift of the protein Ubiquitin taking place during its interaction with gold nanorods (AuNR). In recent years, NMR spectroscopy has been shown to reveal crucial information on the dynamics of protein-nanoparticle interactions, which has important implications for its function^33–35^. It is now understood that such interactions fall in the fast exchange regime of NMR spectroscopy. This implies that when the protein molecules interact with the nanomaterials and form a complex, the resonances of the protein in the NMR spectrum get broader due to the large size of the complex. However, if the experiment is performed such that small amounts of protein is gradually added to the nanomaterial in the NMR tube and the 2D spectrum is recorded after each addition, due to fast exchange the chemical shifts of the protein would appear to move towards the population weighted average value of the free and the bound (complex) forms. When the protein concentration becomes higher than that of the nanomaterial, the resonances get closer to the free (unbound) form. This is shown in Fig. 5 for different additions of Ubiquitin to gold nanorods (the sample preparation is described in the Materials and Methods section). The spectra were collected up to a AuNR:Protein ratio of 0.72:1. Beyond this ratio, the spectrum got significantly broadened and the chemical shifts could not be estimated reliably. Further, due to the large size of the protein-gold nanorod complex, the peaks remained broad throughout the titration due to the population weighted average of transverse relaxation and hence an accurate measurement of their movement is not possible with conventional approaches, because the peaks shift within the linewidths. The ^1^H linewidths observed were 55–60 Hz (0.07 ppm) and chemical shift changes occurred within 2–30 Hz (~0.002–0.04 ppm) with respect to the free protein.

This problem was addressed using the FRI-NMR approach, which enabled the measurement of small shifts occurring during the titration. This is illustrated in Fig. 5b for two cases, wherein the frequencies (chemical shifts) estimated using FRI-NMR are shown superimposed on the Fourier spectrum. As shown in Fig. 5f and g, we could measure changes in ^1^H shifts up to 0.005 ppm (~4 Hz) with respect to the free form. These changes are well within the linewidth (55–60 Hz). Plots for additional residues similar to those shown in Fig. 5f and g are provided in Fig. S8.

The error in ^1^H chemical shift values obtained by FRI-NMR for the low-resolution Ubiquitin-gold nanorod spectrum was estimated by simulating the FID corresponding to the experimental parameter settings. One thousand ^**1**^H FIDs were generated each containing one frequency with 256 complex points, sampled at 12 kHz and corresponding to the observed S/N of ~16 after Fourier transformation (as observed in Fig. 5a). The frequency in each FID was estimated using FRI-NMR and a standard deviation of ~2 Hz (0.0025 ppm) was obtained from 1000 estimations, which was considered as the uncertainty in the ^**1**^H chemical shifts.

Based on the changes in shifts, the interaction strength was quantified via the estimation of the dissociation constant (*K*_D_) and obtained as 27 ± 3 μM (the details of the calculations are given in Section S4 of Supporting Information). The binding constants for proteins with gold nanospheres and nanorods range over several orders of magnitude^36^. This difference is presumably caused by two factors: (1) the differences in energetic contribution from electrostatics, hydrogen bonding, polarizability, hydrophobicity and steric factors and (2) differences between the analytical techniques being used such as analytical centrifugation, fluorescence and NMR spectroscopy, salt concentration and other solvent effects, nature of the nanoparticle shape and capping. However, a commonly observed trend is that particles with a higher surface curvature lead to weaker protein interaction^36^. This would result in a dynamic interaction involving rapid adsorption-desorption of the protein from the surface of the nanorods causing in some cases fast chemical exchange on the NMR time-scale as observed in this study. The functional implication of such an interaction on the function of gold nanorods is currently under investigation.

## Discussion

Spectral resolution in NMR is directly proportional to the duration for which the FID is acquired and the ability to resolve peaks is affected primarily by two factors: (i) the number of frequencies present in a limited spectral region causing overlap of peaks; and (ii) the linewidths of the peaks, which is in turn, dependent on the transverse relaxation rate and/or the number of points used for acquiring the time-domain data^4^. For reducing the number of peaks in a given spectral region, multidimensional NMR is employed to disperse the frequencies, while for addressing the second problem above, approaches such as non-uniform sampling (NUS)^37^, transverse relaxation optimized spectroscopy (TROSY)^8^ or recently proposed pure-shift NMR^38^ can be employed. However, after the data is acquired or reconstructed (in the case of NUS), the only means of resolution enhancement possible are post-acquisition techniques such as apodization, linear prediction, line-shape fitting or spectral deconvolution^6^, which offer limited improvements.

As demonstrated in this paper, linear prediction cannot be of much help beyond the Fourier resolution limit. The alternative approaches of spectral deconvolution and line-shape fitting require prior knowledge of the FID such as the decay rates and number of frequencies present, and are therefore typically used only for analyzing selected spectral peaks of importance for which such information is either available a priori or for which some assumptions could be made. Non-uniform sampling methods achieve high resolution by increasing the length of the FID, while minimizing the acquisition time required for collecting a large number of points by sparse sampling^37^. However, an important requirement in non-uniform sampling is that the signal should last sufficiently long in order to be sampled. If the signal decays rapidly, the resolution is limited by the transverse relaxation. This is exemplified in Fig. S7, wherein spectra with different number of points were reconstructed using NUS. Due to non-Nyquist data acquisition in NUS, proper choices of the sampling points and spectral reconstruction method are necessary to minimize artefacts and preserve the quantitative nature of the peaks. Further, these methods are not currently implemented for 1D NMR experiments, due to the practical difficulty in acquiring non-uniformly sampled data in the direct dimension.

FRI-NMR is not limited by any of the factors mentioned above and allows us to go beyond the resolution limit imposed by the linewidths and enables accurate estimation of the chemical shifts. This is owing to the use of the autocorrelation-based method (described in Section S1). A second-order statistic is used retaining the sum of complex exponentials model of the signal but replacing the noise with its autocorrelation sequence. By assuming that the FIDs are corrupted by additive white Gaussian noise, its autocorrelation sequence is a Kronecker impulse at zero. Hence, the noise has no effect on the samples at locations other than zero. This particular aspect of FRI reconstruction boosts the signal reconstruction accuracy compared with the other methods.

FRI-NMR is particularly useful for signals that decay rapidly. Prior information on the number of peaks or the chemical shifts is not a critical requirement. The underlying assumption is that the signals are made up of damped sinusoids, but this is a perfect match for NMR and not restrictive at all. The samples are acquired on a uniform grid, which does not require special sampling schemes. It is also directly implementable for both 1D and multidimensional NMR experiments. The FRI-NMR reconstruction is linear and the amplitudes of the peaks are not affected allowing the method to be used for quantitative applications. FRI-NMR, which uses only the time-domain signal, could be combined with any of the existing data collection approaches to further improve the resolution. A by-product of this approach is that there is a significant reduction in the number of time-domain points to be collected along the indirect dimension of a multidimensional NMR experiment, resulting in rapid data acquisition. The method could thus also be used for accelerating data collection as a stand-alone approach or in combination with other fast NMR methods^39^.

## Materials and Methods

### Synthesis of gold nanorods (AuNR)

Gold nanorods (AuNR) were prepared based on the seed mediated growth method described by Huang^40^. Briefly, a seed solution was prepared by mixing solution of cetyltrimethylammonium bromide (CTAB) (2.5 mL, 0.20 M) with 2.5 mL of 1 mM HAuCl_4_. To this solution, 0.60 mL of 0.010 M NaBH_4_ was added followed by vigorous stirring, which resulted in the formation of a brownish yellow solution. This seed solution was used for preparation of the gold nanorods. In another flask, CTAB (50 mL, 0.2 M) was added to 2.5 mL of 4 mM AgNO_3_, followed by addition of 50.0 mL of 1 mM HAuCl_4_ to the solution. The solution was gently mixed, followed by addition of 700 µL of 0.078 M Ascorbic acid. The solution changed in color from dark yellow to colourless due to Ascorbic acid, which is a mild reducing agent. Finally, 120 µL of the seed solution was added to the growth solution. These nanorods were aged for 5 h to ensure full growth. After preparation, excess of surfactant, CTAB was removed by centrifuging twice at 12,000 rpm for 10 min, and then re-dispersed in water. The concentration of nanorods in solution was estimated to be 6.0 nM based on a molar extinction coefficient of 3.4 × 10^9^ M^−1^ cm^−1^ ^41^. The gold nanorods were characterized by transmission electron microscopy (TEM), which revealed the length of the rods as ~50 nm and the width as ~20 nm (Fig. 5b).

### Preparation of Ubiquitin

The plasmid (PGLUB) encoding human Ubiquitin was transformed into *E. coli* BL21-DE3 cells. To prepare ^15^N labeled Ubiquitin for NMR studies, cells were grown at 37 °C in a M9 minimal medium consisting of 1 g/L of ^15^N-ammonium chloride and 4 g/L of D-Glucose as the sole source of nitrogen and carbon, respectively. Expression of Ubiquitin was induced by addition of 1.0 mM isopropyl β-D-thiogalactoside (IPTG) at mid-log phase (i.e., when the optical density of cells at 600 nm (O.D_600_) reached ~0.8). Cells were grown for further six hours post-induction, following which they were harvested by centrifugation and solubilized in acetate buffer (5 mM EDTA, 50 mM Na acetate, pH 5). Following sonication, the supernatant containing the protein was loaded onto a pre-equilibrated ion-exchange column (SP Sepharose from GE) and a salt gradient of 0–0.6 M NaCl was used to elute the protein. Following this, the protein was exchanged and concentrated to 1.0 mM in 50 mM Phosphate buffer (95% H_2_O/5%^2^H_2_O; pH 6.0).

### Preparation of p50-NTD

Untagged mouse p50-NTD (residues 39–245) was subcloned in pet11a vector and expressed in *E. coli* (BL21(DE3)). For NMR experiments, the protein fragment was isotopically labeled by growing BL21(DE3) *E. coli* cells expressing p50-NTD in minimal M9 media supplemented with ^15^N-ammonium chloride and ^13^C-glucose as the sole source of nitrogen and carbon, respectively. Protein overexpression was achieved by induction of the cell culture at an optical density at 600 nm of 0.6 by 0.8 mM IPTG followed by overnight incubation with agitation at 25 °C. For purification of p50-NTD, cells were lysed by sonication in lysis buffer (20 mM Tris (pH 7.5), 50 mM NaCl, 1 mM DTT, 0.5 mM EDTA, 0.5 mM PMSF and 10% glycerol) and then centrifuged to remove the insoluble cell debris. p50-NTD remained in the supernatant, which was loaded onto a tandem Q-sepharose followed by SP-sepharose column. The SP-sepharose column was detached, washed with lysis buffer and eluted with NaCl gradient. The elution aliquots containing p50-NTD as detected from 12% SDS-PAGE gel were pooled, concentrated and loaded onto a Superdex-75 column equilibrated with NMR buffer (20 mM Tris (pH 6.8), 50 mM NaCl, 1 mM DTT) for further purification by size-exclusion chromatography. The final NMR sample contained ~0.5 mM of the protein in 600 μL of 95% H_2_O:5%^2^H_2_O.

### NMR spectroscopy

#### 2D [^15^N-^1^H] HSQC spectrum of Ubiquitin and p50-NTD

All NMR data were recorded at 298 K on a BRUKER Avance NMR spectrometer operating at a ^**1**^H resonance frequency of 800 MHz and equipped with a cryogenically cooled triple resonance probe. Chemical shifts were calibrated with respect to 2,2-dimethyl-2-silapentane-5-sulfonate (DSS) (0 ppm) for proton, while ^15^N chemical shifts were calibrated indirectly. The high-resolution 2D HSQC spectrum of Ubiquitin shown in Fig. 3a was acquired with 128 complex points in the^15^N (ω_1_) dimension (spectral width: 2720 Hz) and 1024 complex points in the ^**1**^H (ω_2_) dimension (spectral width: 9600 Hz), with 2 transients and relaxation delay of 1 second between scans, resulting in a measurement time of 9 minutes. The high-resolution 2D HSQC spectrum of p50-NTD shown in Fig. 4a was acquired with 150 complex points in the ^15^N (ω_1_) dimension (spectral width: 2720 Hz) and 1024 complex points in the ^**1**^H (ω_2_) dimension (spectral width: 9600 Hz), with 32 transients and relaxation delay of 1 second between scans, resulting in a measurement time of 150 minutes.

#### Ubiquitin-gold nanorod interaction

To study the interaction of gold nanorods and Ubiquitin, a titration was carried out and chemical shift perturbations were monitored by 2D NMR. For the titration, ^15^N labeled Ubiquitin from a stock solution containing 5 mM of the protein was gradually added to 500 μl of 6.0 nM Gold nanorods taken in the NMR tube. Four additions of the protein were carried out resulting in the following concentrations: 5 μM, 25 μM, 37 μM and 62 μM. These concentrations correspond to AuNR:Protein ratios of 0.72:1, 0.15:1, 0.1:1 and 0.06:1, respectively, assuming that approximately 600 molecules of Ubiquitin adsorb on one gold nanorod particle at any given time (cf. see Section S4 of Supporting Information). Due to the low protein concentration used, SOFAST (band-Selective Optimized Flip-Angle Short-Transient) HMQC^42^ was used for efficient fast data acquisition. The 2D [^15^N, ^1^H]-SOFAST-HMQC was recorded with the ^**1**^H carrier placed at the centre of the amide region (8.5 ppm) and with the ^15^N carrier at 119 ppm. Selective excitation in the amide region was achieved with a 1200 polychromatic pulse with 2.25 ms delay and for inversion R-SNOB pulse was used. The experimental time for each of the HMQC spectra was 11 min with 128 × 1024 complex points along the ^15^N and ^**1**^H dimensions, respectively. However, due to the large size of the complex formed, the FID decayed rapidly within the first 256 complex points in the ^**1**^H dimension. Hence, the data was analysed with the 256 points taken in the ^**1**^H dimension for optimal sensitivity (as done in the case of p50-NTD shown in Fig. 4b). However, for the free Ubiquitin, the full high-resolution spectrum was utilized for comparison.

## Electronic supplementary material

Supplementary Information

## References

[1] Tugarinov V, Hwang PM, Kay LE (2004). Nuclear magnetic resonance spectroscopy of high-molecular weight proteins. Annu. Rev. Biochem..

[2] Duer, M. J. Introduction to solid-state NMR spectroscopy. Blackwell Publishing (2010).

[3] Gowda GAN, Raftery D (2017). Recent advances in NMR-based metabolomics. Anal. Chem..

[4] Cavanagh, J., Fairbrother, W. J., Palmer III, A. G., Rance, M. & Skelton, N. J. Protein NMR Spectroscopy, 2nd ed., Academic Press (2007).

[5] Koehl P (1999). Linear prediction spectral analysis of NMR data. Prog. NMR Spectrosc..

[6] Hoch, J. & Stern, A. NMR Data Processing Wiley (1996).

[7] Waudby CA, Ramos A, Cabrita LD, Christodoulou J (2016). Two dimensional NMR lineshape analysis. Scientific Reports.

[8] Pervushin K, Riek R, Wider G, Wuthrich K (1997). Attenuated T_2_ relaxation by mutual cancellation of dipole–dipole coupling and chemical shift anisotropy indicates an avenue to NMR structures of very large biological macromolecules in solution. Proc. Natl. Acad. Sci. USA.

[9] Parthasarathy S, Nishiyama Y, Ishii Y (2013). Sensitivity and resolution enhanced solid-state NMR for paramagnetic systems and biomolecules under very fast magic angle spinning. Acc. Chem. Res..

[10] Donoho DL (2006). Compressed sensing. IEEE Trans. Inf. Theory.

[11] Donoho DL (2006). For most large underdetermined systems of linear equations, the minimal *l*1-norm solution is also the sparsest solution. Commun. Pure and Appl. Math..

[12] Candes E, Romberg J, Tao T (2006). Robust uncertainty principles: Exact signal reconstruction from highly incomplete frequency information. IEEE Trans. Info. Theory.

[13] Candes E, Wakin M (2008). An introduction to compressive sampling. IEEE Signal Process. Mag..

[14] Vetterli M, Marziliano P, Blu T (2002). Sampling signals with finite rate of innovation. IEEE Trans. Signal Process..

[15] Dragotti PL, Vetterli M, Blu T (2007). Sampling moments and reconstructing signals of finite rate of innovation: Shannon meets Strang-Fix. IEEE Trans. Signal Process..

[16] Tur R, Eldar YC, Friedman Z (2011). Innovation rate sampling of pulse streams with application to ultrasound imaging. IEEE Trans. Signal Process..

[17] Wagner N, Eldar YC, Friedman Z (2012). Compressed beamforming in ultrasound imaging. IEEE Trans. Signal Process..

[18] Pan H, Blu T, Vetterli M (2017). Towards generalized FRI sampling with an application to source resolution in radioastronomy. IEEE Trans. Signal Process..

[19] Bar-Ilan O, Eldar YC (2014). Sub-Nyquist radar via Doppler focusing. IEEE Trans. Signal Process..

[20] Bajwa WU, Gedalyahu K, Eldar YC (2011). Identification of parametric underspread linear systems and super-resolution radar. IEEE Trans. Signal Process..

[21] Castorena J, Creusere CD (2015). Sampling of time-resolved full-waveform LIDAR signals at sub-Nyquist rates. *IEEE Trans*. Geoscience and Remote Sensing.

[22] Seelamantula CS, Villiger ML, Leitgeb RA, Unser M (2008). Exact and efficient signal reconstruction in frequency-domain optical-coherence tomography. J. Opt. Soc. Amer. (A).

[23] Seelamantula CS, Mulleti S (2014). Super-resolution reconstruction in frequency-domain optical-coherence tomography using the finite-rate-of innovation principle. IEEE Trans. Signal Process..

[24] Mulleti S, Shenoy BA, Seelamantula CS (2016). FRI sampling on structured nonuniform grids: Application to super-resolved optical imaging. IEEE Trans. Signal Process..

[25] Dokmanić I, Lu YM (2016). Sampling sparse signals on the sphere: Algorithms and applications. IEEE Trans. Signal Process..

[26] Murray-Bruce J, Dragotti PL (2015). Estimating localized sources of diffusion fields using spatiotemporal sensor measurements. IEEE Trans. Signal Process..

[27] Baechler G, Scholefield A, Baboulaz L, Vetterli M (2017). Sampling and exact reconstruction of pulses with variable width. IEEE Trans. Signal Process..

[28] Pan H, Blu T, Dragotti PL (2014). Sampling curves with finite rate of innovation. IEEE Trans. Signal Process..

[29] Fatemi MM, Amini A, Vetterli M (2016). Sampling and reconstruction of shapes with algebraic boundaries. IEEE Trans. Signal Process..

[30] Roy, R., Paulraj, A. & Kailath, T. ESPRIT - A subspace rotation approach to estimation of parameters of cisoids in noise. *IEEE Trans. Acoustics, Speech, and Signal Process*. **34**, 1340–1342 (1986).

[31] Roy, R. & Kailath, T. ESPRIT - Estimation of signal parameters via rotational invariance techniques. *IEEE Trans. Acoustics, Speech, and Signal Process*. **37**, 984–995 (1989).

[32] Ghosh, G., van Duyne, G., Ghosh, S. & Sigler, P. B. Structure of NF-kappa B p50 homodimer bound to a kappa B-site. *Nature* **373**, 303–310 (1995). 10.1038/373303a0 7530332

[33] Calzolai L, Franchini F, Gilliland D, Rossi F (2010). Protein−nanoparticle interaction: identification of the ubiquitin−gold nanoparticle interaction site. Nano Lett..

[34] Brahmkhatri VP, Chandra K, Dubey A, Atreya HS (2015). An ultrastable conjugate of silver nanoparticles and protein formed through weak interactions. Nanoscale.

[35] Ceccon A, Tugarinov V, AdBax A, Clore GM (2016). Global dynamics and exchange kinetics of a protein on the surface of nanoparticles revealed by relaxation-based solution NMR spectroscopy. J. Am. Chem. Soc..

[36] Boulos SP (2013). The Gold Nanorod-Biology Interface: From Proteins to Cells to Tissue. Curr Phys. Chem..

[37] Mobli M, Hoch JC (2014). Nonuniform sampling and non-Fourier signal processing methods in multidimensional NMR. Prog. NMR Spectrosc..

[38] Zangger K (2015). Pure shift NMR. Prog. NMR Spectrosc..

[39] Atreya HS, Szyperski T (2005). Rapid NMR data collection. Methods Enzymol..

[40] Huang X, El-Sayed IH, Qian W, El-Sayed MA (2006). Cancer cell imaging and photothermal therapy in the near-infrared region by using gold nanorods. J. Am. Chem. Soc..

[41] Orendorff CJ, Murphy CJ (2006). Quantitation of metal content in the silver-assisted growth of gold nanorods. J. Phys. Chem. B.

[42] Schanda P, Kupce E, Brutscher B (2005). SOFAST-HMQC experiments for recording two-dimensional heteronuclear correlation spectra of proteins within a few seconds. J. Biomol. NMR.

