# Supplementary material for: Super-Resolved Nuclear Magnetic Resonance Spectroscopy
Source: Sci Rep. 2017 Aug 29;7:9651. doi: 10.1038/s41598-017-09884-w (PMC5575056; doi:10.1038/s41598-017-09884-w)
Supplement: Supplementary file 1 — Supplementary Information [file 41598_2017_9884_MOESM1_ESM.pdf]

Super-Resolved Nuclear Magnetic Resonance Spectroscopy

Supporting Information

Satish Mulleti[1], Amrinder Singh[2], Varsha Brahmkhatri[2], Kousik Chandra[2], Tahseen Raza[3], Sulakshana Mukherjee[3], Chandra Sekhar Seelamantula[1*], and Hanudatta S. Atreya[2*]

[1]Department of Electrical Engineering, Indian Institute of Science, Bangalore - 560012, India

[2]NMR Research Centre, Indian Institute of Science, Bangalore - 560012, India

[3]Department of Biosciences, Indian Institute of Technology, Roorkee - 247667, India

[*]Corresponding authors' email addresses: chandra.sekhar@ieee.org, hsatreya@sif.iisc.ernet.in

S1. Finite-Rate-of-Innovation-Based Nuclear Magnetic Resonance (FRI-NMR) Spectroscopy

S1.1 Symbols and Notations

The set of real, complex, natural numbers, and integers are represented by $\mathbb{R}$, $\mathbb{C}$, $\mathbb{N}$, and $\mathbb{Z}$, respectively. Matrices and vectors are represented in uppercase and lowercase boldfaced letters, respectively. The matrices $\mathbf{A}^{\mathrm{T}}$, $\mathbf{A}^{*}$, $\mathbf{A}^{\mathrm{H}}$, $\mathbf{A}^{-1}$, and $\mathbf{A}^{\dagger}$ represent transpose, conjugate, Hermitian, inverse, and pseudo-inverse of the matrix $\mathbf{A}$, respectively. The matrices $\mathbf{A}_{\mathrm{F}}$ and $\mathbf{A}_{\mathrm{L}}$ denote the matrices formed by removing the first and last rows, respectively, of the matrix $\mathbf{A}$. The estimate of a vector $\mathbf{a}$ is denoted by $\widehat{\mathbf{a}}$. The notation $[\![N_1, N_2]\!]$ denotes the integer set $\{N_1, N_1+1, N_1+2, \cdots, N_2\}$, where $N_2 > N_1$. The signal $\tilde{f}(t)$ represents the noisy counterpart of $f(t)$. The Fourier transform of $f(t)$ is denoted by $F(\omega)$. The symbol $\mathcal{E}$ denotes the statistical expectation operator.

S1.2 Parameter Estimation of FIDs

A typical one-dimensional free induction decay (FID) could be modeled in the form of a sum of weighted exponentials (SWE), as follows:

$$f(t) = \sum_{\ell=1}^{L} a_\ell \, e^{s_\ell t}, \quad \text{for} \quad t \in [0, T_{\text{obs}}], \tag{S.1}$$

where T_{obs} is the observation time and $s_\ell = -\alpha_\ell + \mathrm{j}\omega_\ell$. In NMR, $\{\alpha_\ell \in \mathbb{R}^+\}$ denote the relaxation rates and $\{\omega_\ell\}$ denote the chemical shifts or frequencies in radians/sec. The goal is to estimate the parameters $\{a_\ell, \alpha_\ell, \omega_\ell\}_{\ell=1}^{L}$ from the samples of $f(t)$ in (S.1), which could be uniform or non-uniform. In this work, we consider uniform samples of the FIDs given by

$$f(nT_s) = \sum_{\ell=1}^{L} a_\ell \, e^{s_\ell n T_s}, \quad \text{for} \quad n \in [\![0, N-1]\!], \tag{S.2}$$

where T_s is the sampling interval or dwell-time. The number of samples are given by $N = \left\lfloor \frac{T_{\text{obs}}}{T_s} \right\rfloor + 1$, where $\lfloor r \rfloor$ denotes the largest integer less than or equal to r. For uniqueness of the estimated ω_ℓs from $f(nT_s)$, the sampling interval T_s should satisfy the inequality $T_s < \dfrac{2\pi}{\omega_{\ell,\max}}$, where $\omega_{\ell,\max}$ denotes the maximum frequency ω_ℓs could achieve.

Among the three unknown parameters of FID signals mentioned above, estimation of ω_ℓs is most important. A standard technique to determine ω_ℓs is to first estimate the Fourier spectrum from $f(nT_s)$ using the discrete-time Fourier transform (DTFT). The frequencies are estimated as the locations of the peaks in the spectrum. The sampled DTFT is computed efficiently using the fast Fourier transform

algorithm. The Fourier approach has two major drawbacks. The first one stems from the fact that due to a finite observation window and non-zero values of α_ℓs, the signal $f(t)$ is not bandlimited. Hence, sampling $f(t)$ at any finite rate $f_s = \frac{1}{T_s}$ will result in aliasing[1,2], which affects the locations of the peaks in the spectra. The Fourier transform of $f(t)$ is given as

$$F(\Omega) = \int_{-\infty}^{+\infty} f(t)\, e^{-\mathrm{j}\Omega t}\, \mathrm{d}t = \sum_{\ell=1}^{L} a_\ell \, \frac{e^{s_\ell T_{\text{obs}} - \mathrm{j}\omega} - 1}{s_\ell - \mathrm{j}\Omega}, \tag{S.3}$$

and it can be shown that aliasing reduces as f_s increases. For $T_s < \frac{2\pi}{\omega_{\ell,\text{max}}}$, the aliasing error depends on T_{obs} and the minimum value of α_ℓ. The other limitation of the Fourier approach is its limited frequency resolution, which is determined by the uncertainty principle. For $\alpha_\ell = 0$, the DTFT of $f(nT_s)$ cannot resolve between two ω_ℓs separated apart by less than $\frac{2\pi}{T_{\text{obs}}}$. In order to resolve closely spaced frequencies, one has to observe the FID for a longer duration, which requires a longer experimentation time. Also, with longer durations, typically, the FIDs decay and noise begins to dominate making the estimates inaccurate and unreliable.

Fourier-based FID analysis does not take into account the SWE model of FIDs. By using this *a priori* signal model, several alternatives have been developed to obtain a resolution higher than that of the Fourier approach for a given number of measurements. A popular approach in the NMR community is linear prediction (LP)[3–7], where each sample $f(nT_s)$ is related to past L contiguous samples by means of a linear combination, that is, there exist L complex numbers c_ℓs such that

$$f(nT_s) = \sum_{\ell=1}^{L} c_\ell\, f(nT_s - \ell T_s), \quad \text{for} \quad n \in [\![L, N-1]\!]. \tag{S.4}$$

The prediction coefficients $\{c_\ell\}$ depend on $\{-\alpha_\ell + \mathrm{j}\omega_\ell\}_{\ell=1}^{L}$. In practice, measurement noise would make the linear combination on the right-hand side of (S.4) an estimate of the left-hand side. By using the linear predictability of $f(nT_s)$, Prony[8] showed that $\{a_\ell, s_\ell\}_{\ell=1}^{L}$ could be computed from $2L$ contiguous values of $f(nT_s)$. Based on Prony's analysis, several LP-based methods have been proposed in both NMR and signal processing literature[3,4,8–15]. There are several variants that use the SWE structure of FID to improve spectral resolution, for example, entropy-based methods[16–18], Bayesian methods[19–21], maximum-likelihood methods[22,23], filter-diagonalization methods[24–26], Padé-Laplace analysis[27,28], etc. We briefly discuss the standard LP technique before proceeding with the development of our technique.

S1.3 Least-Squares Approach with Polynomial Root-Finding

The linear prediction equation in (S.4) could be expressed in matrix form as

$$\underbrace{\begin{pmatrix} f(L-1) & f(L-2) & \cdots & f(0) \\ f(L) & f(L-1) & \cdots & f(1) \\ f(L+1) & f(L) & \cdots & f(2) \\ \vdots & \vdots & \ddots & \vdots \\ f(N-2) & f(N-3) & \cdots & f(N-1-L) \end{pmatrix}}_{\mathbf{F}} \underbrace{\begin{pmatrix} c_1 \\ c_2 \\ c_3 \\ \vdots \\ c_L \end{pmatrix}}_{\mathbf{c}} = \underbrace{\begin{pmatrix} f(L) \\ f(L+1) \\ f(L+2) \\ \vdots \\ f(N-1) \end{pmatrix}}_{\mathbf{f}}. \tag{S.5}$$

For $N \geq 2L$, the matrix $\mathbf{F}$ has rank L and one can uniquely determine $\mathbf{c}$ by solving (S.5) using least-squares regression, and the least-squares solution is $\hat{\mathbf{c}}_{\mathrm{LS}} = (\mathbf{F}^{\mathrm{H}}\mathbf{F})^{-1}\mathbf{F}^{\mathrm{H}}\mathbf{f}$. Alternatively, structured decompositions of $\mathbf{F}$ such as Cholesky decomposition[29], Householder QR decomposition[15], and singular-value decomposition (SVD)[12], could also be deployed to efficiently solve (S.5). Once the c_ℓs are obtained, one could compute the roots of the polynomial $p(z) = \sum_{\ell=0}^{L} c_\ell \, z^\ell$, where $c_0 = 1$, and the roots will be $\{z_\ell = e^{-(\alpha_\ell - \mathrm{j}\omega_\ell)T_s}\}_{\ell=1}^{L}$. This idea forms the basis for several techniques[8,10].

S1.4 LP in the Presence of Noise and Model-Order Selection

In practice, the measurements $\{f(nT_s)\}$ are corrupted by noise. The noisy samples are modeled as

$$\begin{aligned} \tilde{f}(nT_s) &= f(nT_s) + w(n), \\ &= \sum_{\ell=1}^{L} a_\ell \, e^{s_\ell n T_s} + w(n), \end{aligned} \tag{S.6}$$

where $w(n)$s are assumed to be identically distributed, zero-mean, circular white Gaussian random variables[30] with variance σ_w^2. It is also assumed that $w(n)$s are independent of the signal $f(nT_s)$. The noisy samples $\{\tilde{f}(nT_s)\}$ are not linearly predictable, and the prediction coefficients are estimated as the solution to the constrained minimization problem:

$$\min_{\mathbf{c}_a} \quad \left\|[\mathbf{f}\ \mathbf{F}]\mathbf{c}_a\right\|_{\ell_2}^2 \quad \text{subject to} \quad \|\mathbf{c}_a\|_{\ell_2}^2 = 1,$$

where $\mathbf{c}_a \in \mathbb{C}^{L+1}$ and ℓ_2 denotes the Euclidean norm. The solution to the above minimization problem is computed by first performing the SVD of $[\mathbf{f}\ \mathbf{F}]$ and then choosing the right singular vector corresponding to the minimum singular value[31]. This method is effectively linear prediction using SVD (LPSVD)[12]. Several variants of this basic idea are available in the NMR literature.

Another important problem in both NMR and signal processing communities is to estimate the unknown

model order or number of exponentials L from the noisy samples $\{\tilde{f}(nT_s)\}$. For high S/N ratio, there are L distinct large singular values of matrices $\mathbf{F}$ or $[\mathbf{f}\ \mathbf{F}]$ (cf. (S.5)), which could be used as an indicator of the model order. Several techniques have been proposed for estimating the number of exponentials based on information theoretic criteria[32,33], minimum description length (MDL) techniques[34,35], shift-invariant structure[36], Bayesian methods[37], etc.

S1.5 Finite-Rate-of-Innovation Signals and FIDs

Vetterli et al.[31,38] developed a novel paradigm for sampling and reconstructing a class of structured signals that may not be bandlimited, but are characterized by a finite number of parameters per unit interval. Consider a signal of the form $y(t) = \sum_{k\in\mathbb{Z}} d_k\ \psi(t - t_k)$, where the function $\psi(t)$ is known and may not be bandlimited. The signal $s(t)$ is specified by the set of parameters $\{(t_k, d_k),\ k \in \mathbb{Z}\}$. Let $\Gamma_y(\tau_1, \tau_2)$ be the number of free variables of $y(t)$ over $t \in [\tau_1, \tau_2]$. The quantity $R_y = \lim_{\tau\to\infty} \frac{1}{\tau} \Gamma_y\left(-\frac{\tau}{2}, \frac{\tau}{2}\right)$ is termed as the *rate of innovation* of $y(t)$. When R_y is finite, the signal $y(t)$ is referred to as an FRI signal. Vetterli et al. showed that FRI signals, such as stream of Dirac impulses or differential Dirac impulses, piecewise splines, etc., could be reconstructed from samples taken at the rate of innovation using Gaussian or sinc sampling kernels. The reconstruction mechanism is based on high-resolution spectral estimation techniques[30] such as the annihilating filter method[8,31] and estimation of signal parameters via rotational invariance technique (ESPRIT)[39–42]. For a finite duration signal, Vetterli et al. defined the rate of innovation as the average number of free parameters per second, where the average is considered over the duration of the signal.

The FID in (S.1) is specified by $2L$ parameters over the observation interval $[0, T_{\text{obs}}]$: L amplitude values and the set of L exponents $\{-\alpha_\ell + \mathrm{j}\omega_\ell\}_{\ell=1}^{L}$. Hence, the rate of innovation is finite and equal to $\frac{2L}{T_{\text{obs}}}$. Due to the FRI property, FID signals could be reconstructed using a minimum of $2L$ samples. Since the signal structure is known, super-resolution is possible even with a finite number of samples.

S1.6 DEESPRIT – A New Technique for Handling Decaying Exponentials

In the section, we develop a new autocorrelation-based technique for parameter estimation from the FID samples shown in (S.2). In its standard manifestation, ESPRIT is capable of handling only complex sinusoids. On the other hand, FIDs are often decaying and hence, the standard ESPRIT is not directly applicable. We need a counterpart of ESPRIT that is capable of handling decaying exponentials — this is precisely one of our contributions in this paper. The difference between our technique and the other techniques in the signal processing literature that can handle damped exponentials[10,14] is that ours is

based on the autocorrelation. Before going into the details of the proposed method, we briefly discuss standard ESPRIT.

1) ESPRIT for frequency estimation: Consider the problem of estimating the frequencies $\{\omega_\ell\}_{\ell=1}^{L}$ of the complex exponential signal $\tilde{f}(n)$ in noise, given as

$$\tilde{f}(n) = f(n) + w(n) = \sum_{\ell=1}^{L} a_\ell \, e^{\mathrm{j}\omega_\ell n} + w(n), \quad \text{for} \quad n \in [\![0, N-1]\!], \tag{S.7}$$

where $\omega_\ell \in (-\pi, \pi]$ and a_ℓs are independent Gaussian random variables with respective variances $\sigma_{a_\ell}^2$, and $w(n)$s are independent of $f(n)$ and i.i.d. circular Gaussian random variables with zero mean and variance σ_w^2. Let $f_\ell(n) = a_\ell e^{\mathrm{j}\omega_\ell n}$, $\ell \in [\![1, L]\!]$. We have the expectation $\mathcal{E}\{f_\ell(n) f_p^*(m)\} = e^{\mathrm{j}(\omega_\ell n - \omega_p m)} \mathcal{E}\{a_\ell a_p\}$ $= \sigma_{a_\ell}^2 e^{\mathrm{j}(n-m)\omega_\ell} \delta_K[\ell - p]$, for $n, m \in [\![0, N-1]\!]$, and $\ell, p \in [\![1, L]\!]$, where δ_K denotes the Kronecker delta. The expectation is computed jointly over the amplitudes and noise, which are also mutually independent. The autocorrelation sequence of the random process $\tilde{f}(n)$ in (S.7) is given as

$$r_{\tilde{f}\tilde{f}}(n, m) = \mathcal{E}\{\tilde{f}(n)\, \tilde{f}^*(m)\} = \sum_{\ell=1}^{L} \sigma_{a_\ell}^2 \, e^{\mathrm{j}\omega_\ell (n-m)} + \sigma_w^2 \, \delta_K[n-m]. \tag{S.8}$$

Since $r_{\tilde{f}\tilde{f}}(n, m)$ is a function of $n - m$, the process $\tilde{f}(n)$ in (S.7) is stationary. By substituting $k = m - n$, the autocorrelation sequence $r_{\tilde{f}\tilde{f}}(n, m)$ is written as

$$r_{\tilde{f}\tilde{f}}(k) = \mathcal{E}\{\tilde{f}(n)\, \tilde{f}^*(n+k)\} = \sum_{\ell=1}^{L} \sigma_{a_\ell}^2 \, e^{-\mathrm{j}\omega_\ell k} + \sigma_w^2 \, \delta_K[k]. \tag{S.9}$$

By using (S.8), the Mth-order autocorrelation matrix of $\tilde{f}(n)$ is decomposed as

$$\mathbf{R}_{\tilde{f}\tilde{f}} = \mathcal{E}\{\tilde{\mathbf{f}}_{n,M}\, \tilde{\mathbf{f}}_{n,M}^{\mathrm{H}}\} = \begin{pmatrix} r_{\tilde{f}\tilde{f}}(0) & r_{\tilde{f}\tilde{1}}(1) & \cdots & r_{\tilde{f}\tilde{f}}(M-1) \\ r_{\tilde{f}\tilde{f}}(-1) & r_{\tilde{f}\tilde{f}}(0) & \cdots & r_{\tilde{f}\tilde{f}}(M-2) \\ \vdots & \vdots & \ddots & \vdots \\ r_{\tilde{f}\tilde{f}}(1-M) & r_{\tilde{f}\tilde{f}}(2-M) & \cdots & r_{\tilde{f}\tilde{f}}(0) \end{pmatrix},$$

$$= \mathbf{V}\mathbf{A}\mathbf{V}^{\mathrm{H}} + \sigma_w^2 \, \mathbf{I}_M, \tag{S.10}$$

where $\tilde{\mathbf{f}}_{n,M} = [\tilde{f}(n) \;\; \tilde{f}(n+1) \cdots \tilde{f}(n+M-1)]^{\mathrm{T}}$ is an M-length vector, with $M < N$. The components of the matrix $\mathbf{V} \in \mathbb{C}^{M \times L}$ are given as $\mathbf{V}[m, \ell] = e^{-\mathrm{j}\omega_\ell (m-1)}$ for $m \in [\![1, M]\!]$ and $\ell \in [\![1, L]\!]$. The matrix $\mathbf{A} \in \mathbb{R}^{L \times L}$ is a diagonal matrix with entries $\mathbf{A}[\ell, \ell] = \sigma_{a_\ell}^2$, and $\mathbf{I}_M$ is the $M \times M$ identity matrix.

Consider the matrix $\mathbf{U} \in \mathbb{C}^{M \times L}$ whose columns consist of eigenvectors corresponding to the L largest eigenvalues of $\mathbf{R}_{yy}$. It can be shown that, for $M \geq L$, the matrices $\mathbf{U}$ and $\mathbf{R}_{yy}$ have the same range

space[30]. Hence, for $M \geq L$, there exists a nonsingular matrix $\mathbf{P} \in \mathbb{C}^{L\times L}$ such that $\mathbf{U} = \mathbf{V}\,\mathbf{P}$. By using the Vandermonde structure of $\mathbf{V}$, one can write

$$\mathbf{V}_{\mathrm{F}} = \mathbf{V}_{\mathrm{L}}\,\mathbf{Z}, \tag{S.11}$$

where $\mathbf{Z} \in \mathbb{C}^{L\times L}$ is a diagonal matrix with the elements $\mathbf{Z}[\ell,\ell] = e^{-\mathrm{j}\omega_\ell}$. By using the relation $\mathbf{U} = \mathbf{V}\,\mathbf{P}$ and (S.11), one can write

$$\mathbf{U}_{\mathrm{L}} = \mathbf{U}_{\mathrm{F}}\,\mathbf{P}^{-1}\mathbf{Z}\mathbf{P}, \quad \text{or,} \quad \mathbf{P}^{-1}\mathbf{Z}\mathbf{P} = \mathbf{U}_{\mathrm{F}}^{\dagger}\mathbf{U}_{\mathrm{L}}. \tag{S.12}$$

Since $\mathbf{P}^{-1}\mathbf{Z}\mathbf{P}$ has same eigenvalues as $\mathbf{Z}$, which are $\{e^{-\mathrm{j}\omega_\ell}\}_{\ell=1}^{L}$, ω_ℓs can be computed from the eigenvalues of $\mathbf{U}_{\mathrm{F}}^{\dagger}\mathbf{U}_{\mathrm{L}}$, which, in turn, can be computed from the eigenvalues of the autocorrelation matrix $\mathbf{R}_{\tilde{f}\tilde{f}}$.

2) DEESPRIT for parameter estimation of damped exponentials: Consider the samples $\tilde{f}(n)$ in (S.6) for $n \in [\![0, N-1]\!]$. We assume that a_ℓs are independent Gaussian random variables with respective variances $\sigma_{a_\ell}^2$. The correlation between the random variables $f_\ell(nT_s) = a_\ell\, z_\ell^n$ and $f_p(mT_s) = a_p\, z_p^m$ is given as

$$\mathcal{E}\{f_\ell(nT_s)\, f_p^*(mT_s)\} = \mathcal{E}\{a_\ell\, a_p\} z_\ell^n z_p^{*m} = \sigma_{a_\ell}^2\, z_\ell^n z_\ell^{*m} \delta_K[\ell - p], \tag{S.13}$$

where $z_\ell = e^{-(\alpha_\ell - \mathrm{j}\omega_\ell)T_s}$, and the expectation is computed over the amplitudes. By using (S.13), the autocorrelation sequence of the random process $\tilde{f}(nT_s)$ in (S.6) is written as

$$\begin{aligned} r_{\tilde{f}\tilde{f}}(nT_s, mT_s) &= \mathcal{E}\{\tilde{f}(nT_s)\,\tilde{f}^*(mT_s)\}, \\ &= \sum_{\ell=1}^{L} \sigma_{a_\ell}^2\, z_\ell^n\, \bar{z}_\ell^m + \sigma_w^2 \delta_K[n-m], \quad \text{for} \quad n, m \in [\![0, N-1]\!], \end{aligned} \tag{S.14}$$

where the expectation is taken jointly over the amplitudes and noise. Since $|z_\ell|$ is not necessarily equal to unity, $r_{\tilde{f}\tilde{f}}(nT_s, mT_s)$ cannot be written as a function of only $n - m$. This is a consequence of the nonstationarity of the random process $f(nT_s)$ in (S.6) unlike the process in (S.7), which is stationary. Due to the nonstationarity, we cannot directly construct a matrix of the form given in (S.10).

Consider $f(nT_s)$ in (S.6), and the Mth-order autocorrelation matrix $\mathbf{R}_{\tilde{f}\tilde{f}}(nT_s) = \mathcal{E}\{\tilde{\mathbf{f}}_{n,M}\,\tilde{\mathbf{f}}_{n,M}^{\mathrm{H}}\}$, where $\tilde{\mathbf{f}}_{n,M} = [\tilde{f}(nT_s)\;\; \tilde{f}((n+1)T_s) \cdots \tilde{f}((n+M-1)T_s)]^{\mathrm{T}}$. The index n is chosen such that $n+i \in [\![0, N-1]\!]$

for $i \in [\![0, M-1]\!]$ for a given $M < N$. The $(k_1, k_2)^{\text{th}}$ element of $\mathbf{R}_{\tilde{f}\tilde{f}}(nT_s)$ is

$$\begin{aligned}\mathbf{R}_{\tilde{f}\tilde{f}}(nT_s)[k_1, k_2] &= \mathcal{E}\{\tilde{f}((n+k_1)T_s)\tilde{f}^*((n+k_2)T_s)\},\\ &= \sum_{\ell=1}^{L} \sigma_{a_\ell}^2 \, |z_\ell|^{2n} \, z_\ell^{k_1} \, \bar{z}_\ell^{k_2} + \sigma_w^2 \delta_K[k_1 - k_2].\end{aligned} \tag{S.15}$$

To remove the dependency of the matrix $\mathbf{R}_{\tilde{f}\tilde{f}}(nT_s)[k_1, k_2]$ on n, we construct the matrix $\mathbf{R}^a_{\tilde{f}\tilde{f}}$, which is computed as $\mathbf{R}^a_{\tilde{f}\tilde{f}} = \dfrac{1}{N}\displaystyle\sum_{n=0}^{N-1} \mathbf{R}_{\tilde{f}\tilde{f}}(nT_s)$. The elements of $\mathbf{R}^a_{\tilde{f}\tilde{f}}$ are given as

$$\begin{aligned}\mathbf{R}^a_{\tilde{f}\tilde{f}}[k_1, k_2] &= \frac{1}{N}\sum_{n=0}^{N-1} \mathcal{E}\{\tilde{f}((n+k_1)T_s)\tilde{f}^*((n+k_2)T_s)\},\\ &= \sum_{\ell=1}^{L} b_\ell \, z_\ell^{k_1} \, \bar{z}_\ell^{k_2} + \sigma_w^2 \delta_K[k_1 - k_2],\end{aligned} \tag{S.16}$$

where $b_\ell = \dfrac{\sigma_{a_\ell}^2}{N}\displaystyle\sum_{n=1}^{N-1} |z_\ell|^{2n} = \dfrac{1}{N}\sigma_{a_\ell}^2 \dfrac{1 - |z_\ell|^{2N}}{1 - |z_\ell|^2}$. Further, $\mathbf{R}^a_{\tilde{f}\tilde{f}}$ can be decomposed as $\mathbf{R}^a_{\tilde{f}\tilde{f}} = \mathbf{Z}\mathbf{B}\mathbf{Z}^{\mathrm{H}} + \sigma_w^2 \, \mathbf{I}_M$, where

$$\mathbf{Z} = \begin{pmatrix} 1 & 1 & \cdots & 1 \\ z_1 & z_2 & \cdots & z_L \\ z_1^2 & z_2^2 & \cdots & z_L^2 \\ \vdots & \vdots & \ddots & \vdots \\ z_1^{M-1} & z_2^{M-1} & \cdots & z_L^{M-1} \end{pmatrix} \in \mathbb{C}^{M-1 \times L}.$$

The matrix $\mathbf{R}^a_{\tilde{f}\tilde{f}}$ has a form similar to that of $\mathbf{R}_{\tilde{f}\tilde{f}}$ in (S.10). Now, one could apply the matrix decompositions developed for classical ESPRIT to $\mathbf{R}^a_{\tilde{f}\tilde{f}}$.

In practice, the autocorrelation matrix $\mathbf{R}^a_{\tilde{f}\tilde{f}}$ is estimated from the noisy samples $\{\tilde{f}(n)\}_{n=0}^{N-1}$ as

$$\widehat{\mathbf{R}}^a_{\tilde{f}\tilde{f}} = \frac{1}{N}\sum_{n=0}^{N-M} \tilde{\mathbf{f}}_{n,M} \, \tilde{\mathbf{f}}_{n,M}^*. \tag{S.17}$$

S1.7 Amplitude Estimation in FRI-NMR

Once the frequencies are estimated, the amplitudes $\{a_\ell\}_{\ell=1}^L$ are estimated by applying linear least-squares regression on the noisy samples $\{\tilde{f}(n)\}_{n=0}^{N-1}$. The estimated amplitudes are given by

$$\widehat{\mathbf{a}} = \widehat{\mathbf{Z}}^\dagger \, \tilde{\mathbf{f}}_{0,N}, \tag{S.18}$$

where

$$\widehat{\mathbf{Z}} = \begin{pmatrix} 1 & 1 & \cdots & 1 \\ \widehat{z}_1 & \widehat{z}_2 & \cdots & \widehat{z}_L \\ \widehat{z}_1^2 & \widehat{z}_2^2 & \cdots & \widehat{z}_L^2 \\ \vdots & \vdots & \ddots & \vdots \\ \widehat{z}_1^{N-1} & \widehat{z}_2^{N-1} & \cdots & \widehat{z}_L^{N-1} \end{pmatrix} \in \mathbb{C}^{N-1\times L}, \quad \text{and} \quad \widehat{\mathbf{a}} = \begin{pmatrix} \widehat{a}_1 \\ \widehat{a}_2 \\ \widehat{a}_3 \\ \vdots \\ \widehat{a}_L \end{pmatrix}.$$

S1.8 Parameter Estimation Using FRI-NMR – A Simulation

The first simulation is designed to demonstrate the parameter estimation capability of the FRI-NMR technique over a smaller number of measurements and compare with the Fourier method. We considered an FID with $N = 2000$ samples (measured at 12 kHz) of a three-component signal ($L = 3$) where the frequencies were chosen as 3000 Hz, 3015 Hz, and 3050 Hz. The corresponding damping coefficients and amplitudes were selected as $\{3, 2, 4\}$ and $\{6, 4, 2\}$, respectively. The samples were corrupted by circular white complex Gaussian noise with zero mean and variance determined according to a desired S/N ratio. The parameters are estimated by applying the FRI-NMR and the Fourier method by using the first 600 samples of the total samples. The samples are apodized with a $\cos(30)$ window function shown below.

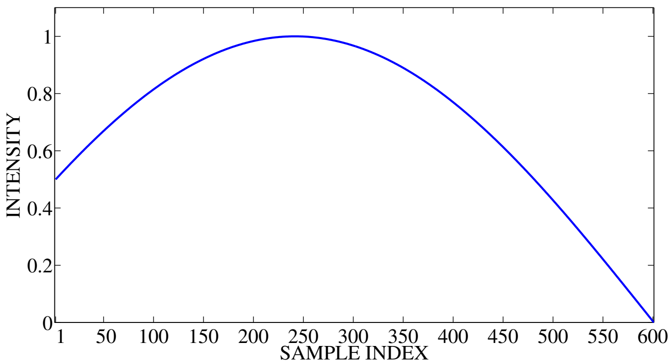

$\cos(30)$apodization window function.

The Fourier spectra are measured by zero padding the samples to extend its length to 2^{16}. The parameters are estimated over 200 independent trials. Figure S1(a) shows one realization of the magnitude spectrum of the truncated noisy FID. The average S/N of the magnitude spectra of the truncated FID is 10. The averaged estimates of the frequencies, damping coefficients, and amplitudes are $[3001, 3013.4, 3050]$ Hz, [8, 4.3, -1], and [6.7 3.75, 1.68], respectively. Figure S1(b) shows a zoomed-in part of the spectrum corresponding to full FID (shown in green) and truncated FID (in black). To facilitate visual comparison, the Fourier spectra are normalized to have maximum value equal to the maximum amplitude of the FID

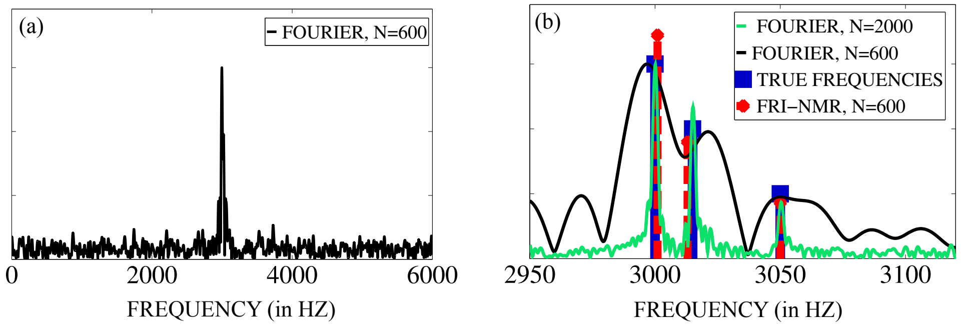

Fig. S1: A visual comparison of parameter estimation performance: (a) Magnitude spectra of the noisy FID using 600 samples and average S/N equal to 10; (b) Zoomed-in part of the spectrum together with the ground-truth frequencies on which the DEESPRIT estimates are superimposed. FRI-NMR with the help of DEESPRIT is able to achieve frequency and amplitude estimates comparable with those of the Fourier method by using only 30% of the samples.

components, which is 6 in this experiment. In Fig. S1(b), the true frequencies are marked by Dirac impulses with true amplitudes (stem plots in blue). The frequencies estimated by FRI-NMR are shown as Dirac impulses in red color with their amplitudes equal to the corresponding estimated values. We observe that by using 600 samples, the Fourier method gives broad lines with peak maxima shifted away from the true frequencies, whereas the FRI-NMR method gives frequency estimates closer to the actual values. For S/N $= 10$, the estimates of the damping coefficients are not accurate. However, the amplitude estimates are closer to the true values as shown in Fig. S1(b). The average frequency and amplitude estimated by applying FRI-NMR on 600 noisy samples are comparable with those obtained using the Fourier method with 2000 samples.

S1.9 Accuracy of Frequency Estimation in FRI-NMR as Function of Damping and Amplitudes

In this section, we analyze experimentally the accuracy of frequency estimation (measured in Hz) as a function of the damping factor α and amplitude a. We consider 256 samples of a single-component FID with frequency and sampling rate of 500 Hz and 1500 Hz, respectively. The signal is corrupted by zero-mean, additive white Gaussian noise (AWGN) with a fixed standard deviation of 2.5. The frequency is estimated by FRI-NMR as the amplitude is increased from 1 to 5 in steps of 1 and for $\alpha = 1, 2, \cdots, 9, 10,$ and 20. For a given amplitude and damping, we computed average S/N and root mean-squared error in frequency estimation over 500 independent noise realizations.

The computed average S/N is shown in Figure S2(a) and RMSE in Figure S2(b). As α increases, the signal decays faster, and consequently, the S/N decreases, which results in a large RMSE. As amplitude increases, S/N increases and hence RMSE decreases.

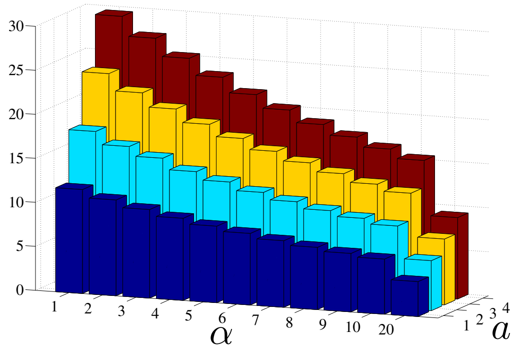

(a) Average S/N

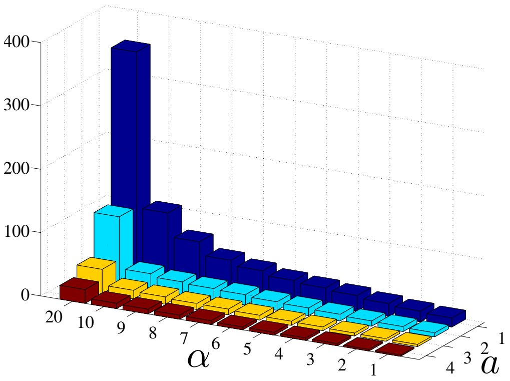

(b) RMSE in Estimation of Frequency (in Hz)

Fig. S2: Accuracy of frequency estimation (shown in b) in FRI-NMR as function of damping α and amplitude a; RMSE increases as α increases and it decreases with increase in amplitude.

S2. COMPARISON OF FRI-NMR WITH LPSVD AND FOURIER METHODS

The goal of this experiment is to asses the resolution capability of the FRI-NMR method and compare it with that of the LPSVD (cf. Section S1) and Fourier methods. We considered $N = 600$ noisy FID samples (cf. (S.6)) for $L = 2$ with $a_1 = a_2 = 1$, $\alpha_1 = \alpha_2 = 3$. The frequencies of the two components are $F_1 = 5000$ Hz and $F_2 = 5000 + \Delta F$ Hz, where ΔF represents the frequency separation. The FID was sampled at 12 kHz and corrupted by zero mean additive white Gaussian noise (AWGN) to simulate a noisy measurement, with a variance σ_w^2 chosen to achieve a given S/N. The samples are apodized by using $\cos(30)$ window function. To compute the Fourier spectra, the samples are zero-padded to have a length of 2^{17}. We chose similar amplitudes and damping factors for the two FID components such that they have equal S/N. The performance of the technique in resolving the two frequencies is assessed by varying ΔF below the resolution limit given by $\frac{1}{T_{\text{obs}}} \leq \frac{1}{(N-1)T_s} = 20.034$ Hz.

We observed that for a given S/N, ΔF, and noise realization, the LPSVD and FRI-NMR methods may not always estimate two distinct frequencies. Based on the estimates $\hat{F}_1$ and $\hat{F}_2$, we classify the outcome of a trial for a given method as belonging to one of the following three categories: (i) *Resolved* if $\hat{F}_1$ and $\hat{F}_2$ are distinct and lie within the frequency interval $[F_1 - 50, F_2 + 50]$; (ii) *Unresolved* if a single frequency, either $\hat{F}_1$ or $\hat{F}_2$, is estimated to lie within the interval $[F_1 - 50, F_2 + 50]$ Hz; (iii) *Missed* if none of the estimated frequencies belong to the interval $[F_1 - 50, F_2 + 50]$ Hz. We estimated the frequencies by applying Fourier, LPSVD, and FRI-NMR methods for S/N $= 7.5, 10$ and 15, and for ΔF increasing from 5 Hz to 25 Hz in steps of 2.5 Hz. In Fourier-based estimation, the frequencies are estimated from the peaks of the real part of the spectra. For each S/N and ΔF, the frequencies are estimated for 1000 independent noise realizations and the results are categorized as explained above. Figure S3 shows bar plots that indicate the number of times a given method resolves, misses, or does not resolve the frequencies, out of a total of 1000 trials. The classification metric shown in these bar plots show the resolution capability but does not quantify the error in the estimation. To quantify the accuracy of the methods, for a given S/N and ΔF, we compute normalized bias, root-mean variance, and root-mean-squared error (RMSE) in Hz. These three metrics are computed as

$$\text{BIAS} = \mathcal{E}_s\left\{0.5(F_1 - \hat{F}_1) + 0.5(F_2 - \hat{F}_2)\right\}/\Delta F, \tag{S.19}$$

$$\text{VAR} = \sqrt{\mathcal{E}_s\left\{0.5\left(\hat{F}_1 - \mathcal{E}_s\{\hat{F}_1\}\right)^2 + 0.5\left(\hat{F}_2 - \mathcal{E}_s\{\hat{F}_2\}\right)^2\right\}}/\Delta F, \tag{S.20}$$

$$\text{RMSE} = \sqrt{\mathcal{E}_s\left\{0.5\left(F_1 - \hat{F}_1\right)^2 + 0.5\left(F_2 - \hat{F}_2\right)^2\right\}}/\Delta F, \tag{S.21}$$

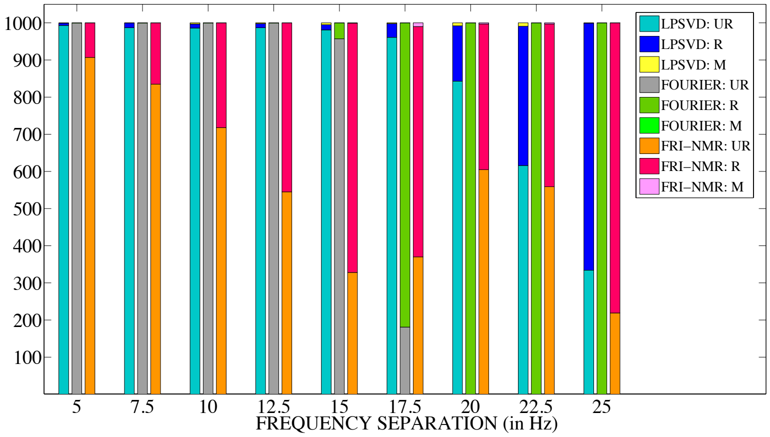

(a) S/N=7

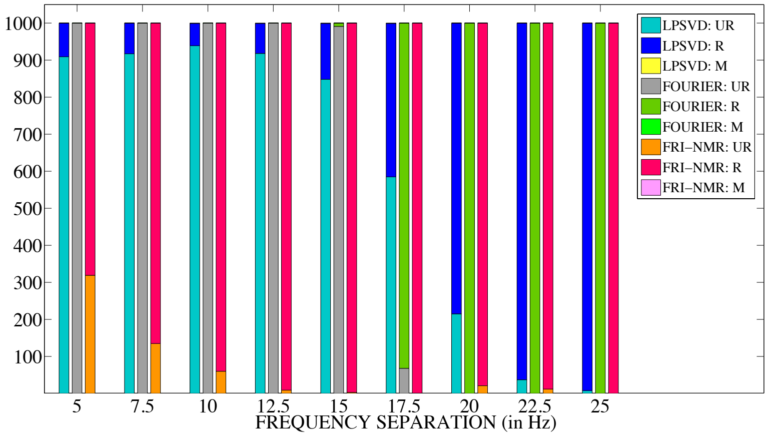

(b) S/N=10

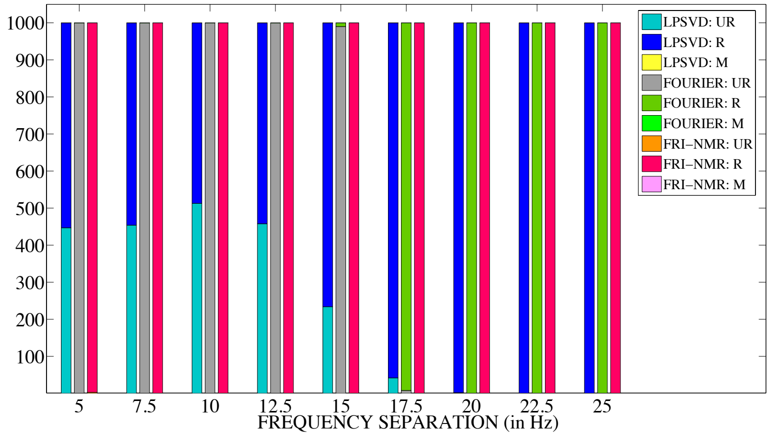

(c) S/N=15

Fig. S3: A comparison of consistency of frequency resolution ability of different methods in terms of the number of times the estimated frequencies were resolved (‘R’), unresolved (‘UR’), and missed (‘M’), out of a total of 1000 independent noise realizations of noise for a given S/N and ΔF. For each triplet stacked bars, the left, middle, and right bars correspond to LPSVD, Fourier, and FRI-NMR, respectively.

respectively, where $\hat{F}_1$ and $\hat{F}_2$ denote the estimated values of F_1 and F_2, respectively, and $\mathcal{E}_s$ denotes the sample mean measured over 1000 independent realizations of noise. In order to obtain meaningful BIAS, VAR, and RMSE when the frequencies are unresolved, that is when both F_1 and F_2 have only one estimate that lies within the interval $[F_1 - 50, F_2 + 50]$ Hz, we assign the same value for both $\hat{F}_1$ and $\hat{F}_2$ and compute these metrics as shown in Figure S4. Any conclusion on the results in this analysis should be made by simultaneously analyzing Figures S3 and S4.

To illustrate further, consider the case where S/N $=$ 10. By applying apodization and by using dissipative spectra (real part of the Fourier spectra), in Figure S3, we observe that FRI-NMR is able to resolve frequencies at 5 Hz, whereas the Fourier method is able to distinguish frequencies separated by 17.5 Hz. Comparing LPSVD and FRI-NMR, we find that both techniques are able to estimate frequencies separated by 5 Hz but LPSVD is able to resolve frequencies only 10% of the time compared with FRI-NMR, which is able to resolve frequencies about 70% of the time.

Further, comparing the plots in Figure S4, the flat region in the bias and MSE curves of the Fourier plots signify that the Fourier method is unable to resolve the frequencies and gives only a single peak about the average $(F_1 + F_2)/2$. Hence, the error is constant. As the frequency separation increases beyond 17.5 Hz, the Fourier method is able to resolve the frequencies, which is also shown in Figure S3 – this results in reduction in error. The Fourier method doesnt have any case where it misses, whereas, both LPSVD and FRI-NMR method have few cases where none of the estimated frequencies fall in the range of $[F_1 + 50, F_2 - 50]$. Such outliers result in large bias and MSE. For example, at S/N $= 10$, for $\Delta F \leq 7.5$ Hz, both FRI-NMR and LPSVD have outliers due to misses, which results in a large bias and MSE.

These experiments showed that FRI-NMR is capable of resolving closely spaced frequencies, which are not resolved by the standard Fourier method. Further, the bias and MSE in frequency estimation are smaller in case of FRI-NMR compared with the Fourier method, when the components are separated by $10 - 17.5$ Hz.

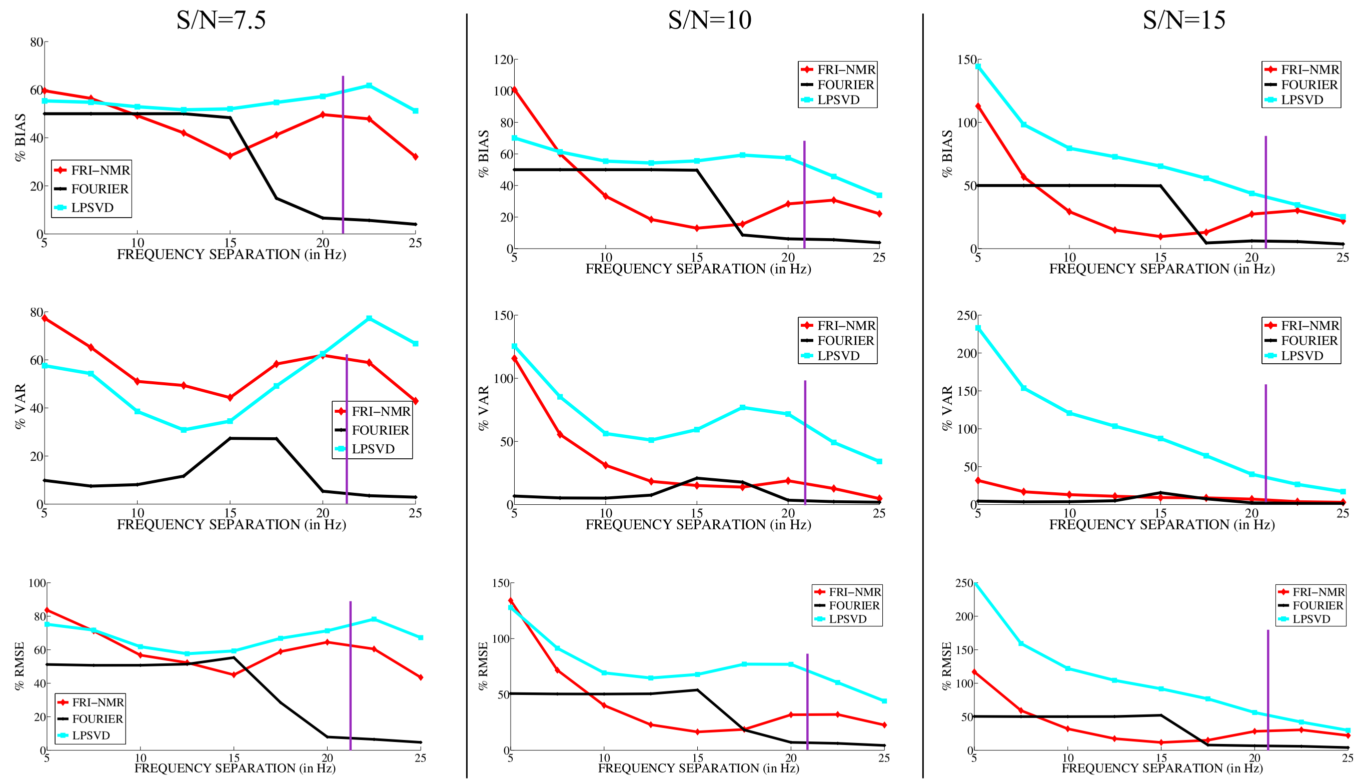

Fig. S4: Performance comparison of different methods in terms of % BIAS, %VAR, and %NMSE. For all S/N and ΔF, LPSVD method has BIAS and NMSE that are $0 - 38\%$ larger than Fourier and FRI-NMR methods. FRI-NMR has $0 - 15\%$ lesser BIAS and NMSE compared with the Fourier method at S/N $= 10$ and 15 for different ΔF values.

S3. Model Order Selection for Experimental Data

The FRI-NMR method requires specification of the model order L. Typically, in NMR spectroscopy, the maximum number of exponentials or chemical shifts present in a sample is known a priori. This information could be used in FRI-NMR to estimate the chemical shifts. To have a more precise estimate of the model order, we used singular values of the autocorrelation matrix (S.16). Figure S5 shows the singular values of 24 kDa P50-NTD FIDs of autocorrelation matrices where $M = 20$ and $N = 256$. In these four instances, we observe that few singular values have larger magnitudes, which correspond to signal components of noisy FIDs. For example, in Figs. S5(a), S5(b), S5(c), and S5(d) there are one, three, two, and four relatively larger singular values, respectively, which can be used as the model order in each case.

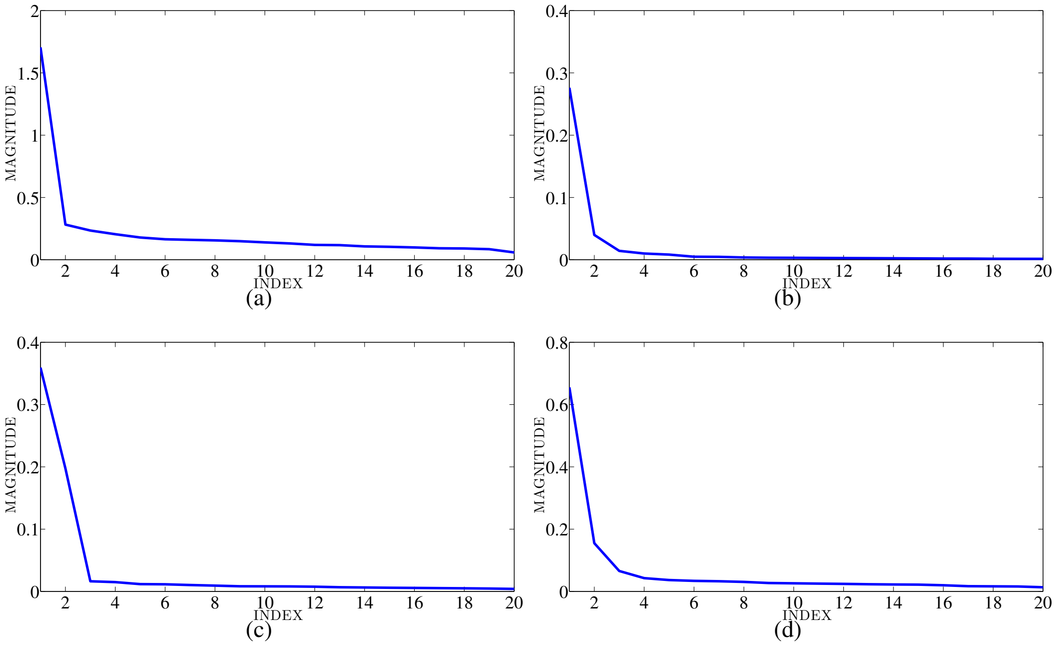

Fig. S5: Model order selection from the singular values of the autocorrelation matrix. The plots show the singular values of the corresponding autocorrelation matrix for four instances of the 24 kDa P50-NTD FIDs (cf. Fig. 4 in the main document). The number of large singular values in these examples 1, 3, 2, and 4, respectively and are used as respective model orders in FRI-NMR estimation.

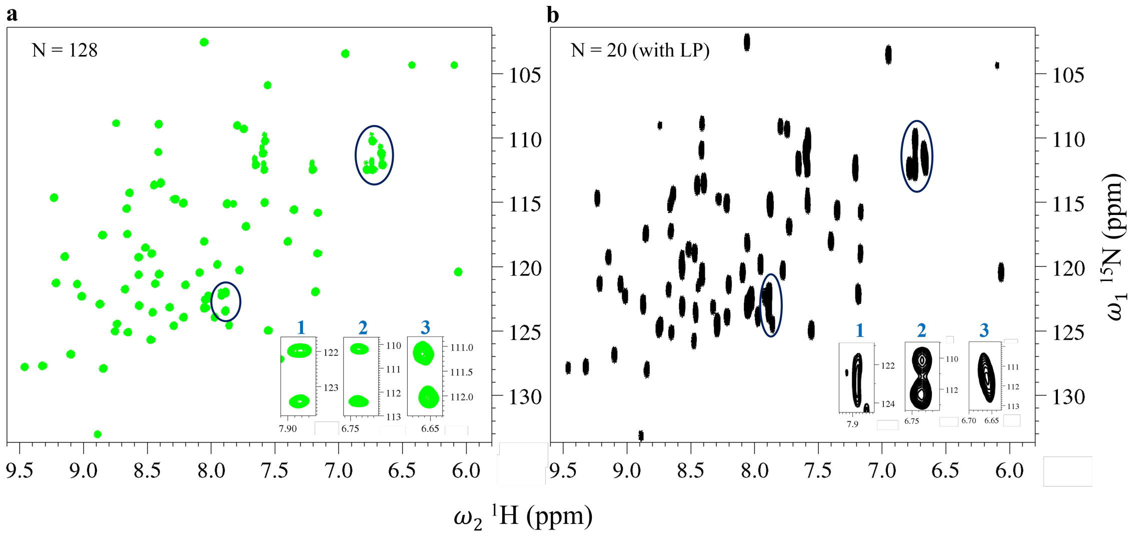

Fig. S6: (a) The 2D [^{15}N-^{1}H] HSQC spectrum of Ubiquitin acquired with 128 complex points along the indirect dimension (indicated as $N = 128$). (b) A low-resolution spectrum obtained from (a) by considering the first 10 complex points in the FID along the ^{15}N dimension (ω_1) and extending it by another 10 points by linear prediction resulting in a total of 20 complex points. The three regions analyzed in Fig. 5 are also shown magnified in the insets. Thus, both Fourier transformation with $N = 20$ and doubling the FID in the case of linear prediction are not capable of resolving the spectral peaks.

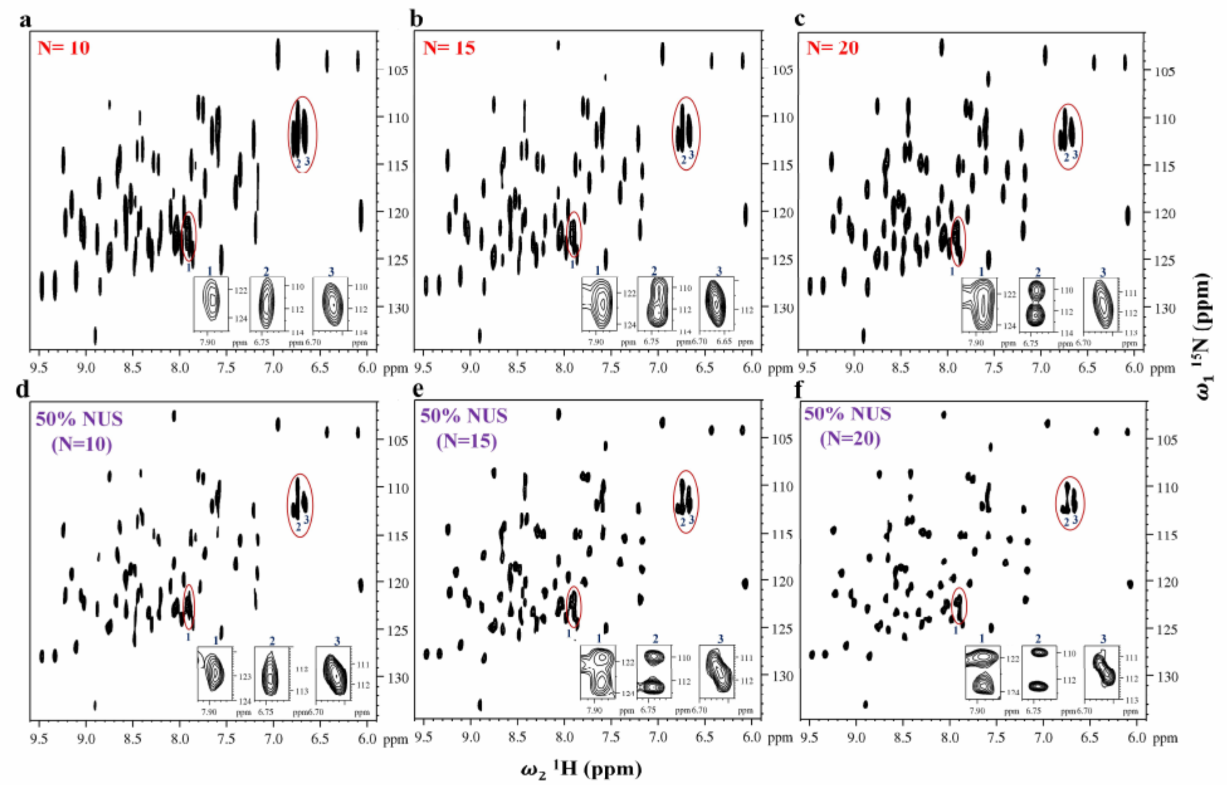

Fig. S7: (a)-(c) An experimental dataset was acquired on Ubiquitin wherein the FID was collected for the first 10, 15 and 20 complex points in the ^{15}N dimension and Fourier transformed. (d)-(f) A second set of FIDs were sampled at 50% of the points using a non-uniformly sampled schedule based on Poisson gap sampling implemented in the Iterative Soft Thresholding @ Harvard Medical School (HMS-IST) program available at: For 10 complex points 50% NUS schedule was: 0, 1, 2, 5, 9; For 15 complex points: 0, 1, 2, 3, 4, 7, 10, 13; For 20 complex points: 0, 1, 2, 3, 6, 8, 11, 12, 14, 18. The 2D spectrum was reconstructed using the IST-HMS method. It is evident that with NUS the peaks are not resolved when 10 or 15 complex points are chosen.

S4. Estimation of the Dissociation Constant (K_D) of Ubiquitin Gold-Nanorod Interaction

The dissociation constant for the Ubiquitin Gold-nanorod (AuNR) interaction was determined assuming a fast exchange. First, the number of Ubiquitin molecules binding one nanorod was estimated based on the surface area of the nanorods, which was calculated based on its dimensions observed in the transmission electron microscopy (TEM) (Fig. 5b of the main document) and depicted below.

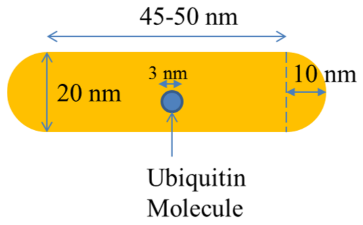

Assuming the Ubiquitin molecules are uniformly and tightly packed on the surface of the nanorod, the total surface area was divided by the area occupied by one Ubiquitin molecule. The area occupied by one Ubiquitin molecule on the surface was conservatively estimated as a circle having a radius corresponding to the hydrodynamic radius of Ubiquitin (1.5 nm). Using this approach, the total number of Ubiquitin molecules adsorbed on the surface of the gold nanorod was approximately estimated as 600.

Next, we assumed that all the binding sites on the nanorods are independent of each other and the binding of the peptide to one site does not affect its binding to another site on the AuNR surface. The dissociation constant (K_D) was then obtained by fitting Equation (S.22) to the chemical shift data obtained from the titration experiment, which is valid for fast exchange and for a system with multiple binding sites for the ligand[43]. Here, the protein represents the ligand and the nanorod represents the macromolecule on which binding takes place:

$$\delta_{\text{obs}} - \delta_{\text{free}} = \frac{\delta_{\max}}{2\,[\text{Ubq}]_{\text{total}}}\Bigg((N_{\text{ubq}}\,[\text{AuNR}]_{\text{total}} + [\text{Ubq}]_{\text{total}} + K_D) - \sqrt{(N_{\text{ubq}}\,[\text{AuNR}]_{\text{total}} + [\text{Ubq}]_{\text{total}} + K_D)^2 - 4N_{\text{ubq}}\,[\text{AuNR}]_{\text{total}}[\text{Ubq}]_{\text{total}}}\Bigg), \qquad \text{(S.22)}$$

where δ_{obs} and δ_{free} denote the observed chemical shifts of the bound peptide and free peptide, respectively. The symbols $[\text{AuNR}]_{\text{total}}$ and $[\text{Ubq}]_{\text{total}}$ represent the total concentration of AuNR and Ubiquitin at a given point during the titration, respectively. In (S.22), we have $\delta_{\max} = \delta_{\text{bound}} - \delta_{\text{free}}$, where δ_{bound} is the chemical shift in the completely bound form. The $\delta_{\max}$ is obtained as part of the fitting procedure[43]. Using this equation, the K_D was estimated as $27 \pm 3\,\mu$M.

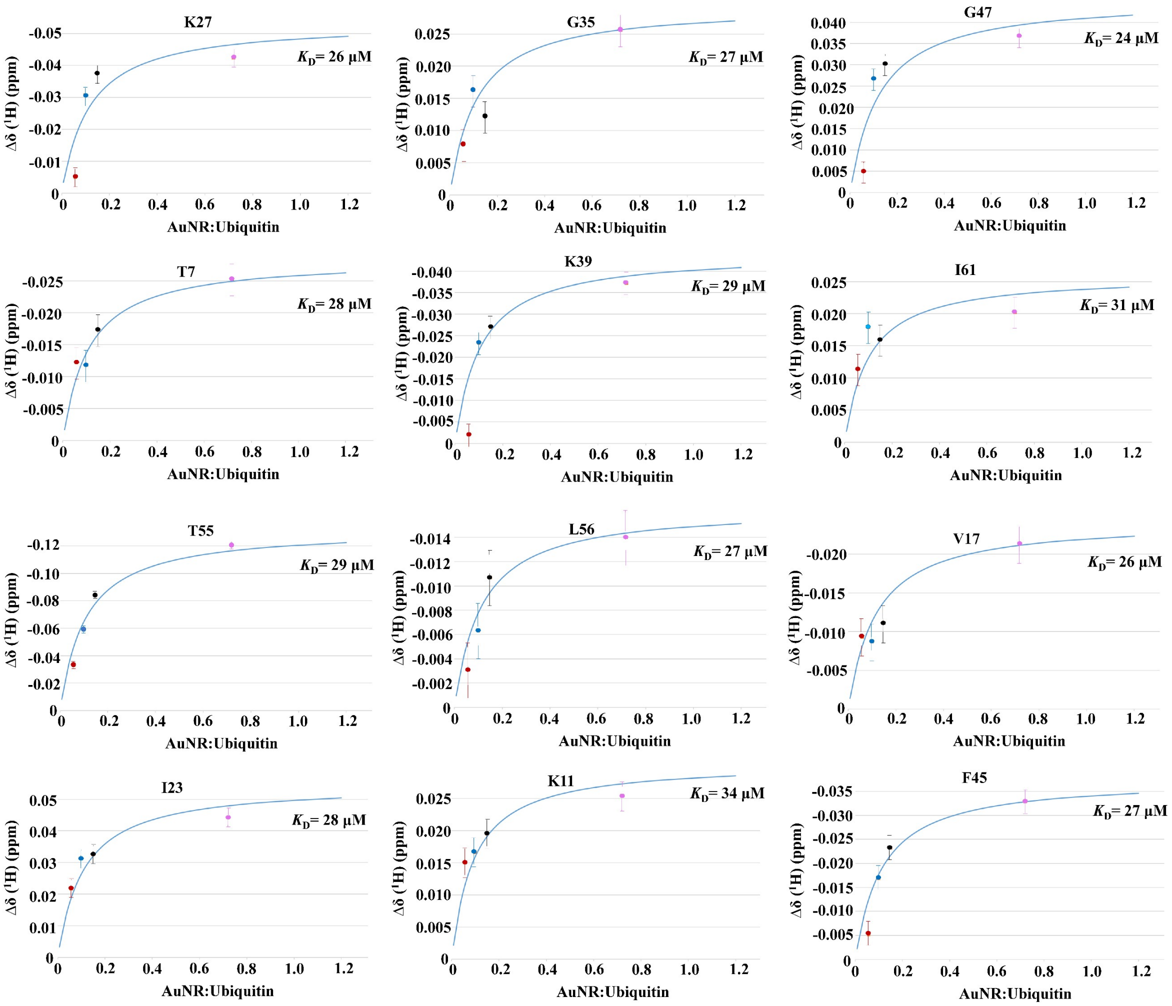

Fig. S8: Plots of ^{1}H chemical shift changes in Ubiquitin upon addition to the gold nanorod sample for a few residues with respect to the free protein (see Figure 4a of Main text). The different concentrations of ubiquitin at which the chemical shift changes were measured from 2D [^{15}N-^{1}H] HSQC spectrum are: ● 5 μM (AuNR: Ubiquitin = 0.72); ● 25 μM (AuNR:Ubiquitin = 0.15); ● 35 μM (AuNR:Ubiquitin = 0.1); ● 62 μM (AuNR:Ubiquitin = 0.06); The number of ubiquitin molecules adsorbed on the gold-nanorod at any given time was taken as 600 (see Section S5) and the dissociation constant (K_D) indicated for each residue was obtained by fitting the shifts to the equation shown in Section S5 corresponding to fast exchange. The error bars correspond to the estimated uncertainty in the measurement of ^{1}H shifts (0.0025 ppm) as described in the main text.

References

[1] Shannon, C. E. A mathematical theory of communication. *The Bell Syst. Tech. J.* **27**, 623–656 (1948).

[2] Nyquist, H. Certain topics in telegraph transmission theory. *Trans. American Inst. Elect. Eng.* **47**, 617–644 (1928).

[3] Stephenson, D. S. Linear prediction and maximum entropy methods in NMR spectroscopy. *Prog. Nucl. Mag. Reson. Spectrosc.* **20**, 515 – 626 (1988).

[4] Koehl, P. Linear prediction spectral analysis of NMR data. *Prog. Nucl. Mag. Reson. Spectrosc.* **34**, 257 – 299 (1999).

[5] Makhoul, J. Linear prediction: A tutorial review. *Proc. IEEE* **63**, 561–580 (1975).

[6] Vaidyanathan, P. P. *The Theory of Linear Prediction*. Synthesis Lectures on Engineering Series (Morgan & Claypool, 2008).

[7] Markel, J. D. & Gray, A. H. *Linear Prediction of Speech*. Communication and Cybernetics (Springer-Verlag, 1976).

[8] DeProny, G. R. Essai experimental et analytique: Sur les lois de la dilatabilité de fluides élastiques et sur celles de la force expansive de la vapeur de l'eau et de la vapeur de l'alcool, à différentes températures. *J. de l'Ecole polytechnique* **1**, 24–76 (1795).

[9] Tufts, D. W. & Kumaresan, R. Estimation of frequencies of multiple sinusoids: Making linear prediction perform like maximum likelihood. *Proc. IEEE* **70**, 975–989 (1982).

[10] Kumaresan, R. & Tufts, D. Estimating the parameters of exponentially damped sinusoids and pole-zero modeling in noise. *IEEE Trans. Acoustics, Speech, and Signal Process.* **30**, 833–840 (1982).

[11] Barkhuijsen, H., de Beer, R., Bovée, W. M. M. J. & van Ormondt, D. Retrieval of frequencies, amplitudes, damping factors, and phases from time-domain signals using a linear least-squares procedure. *J. Mag. Reson.* **61**, 465 – 481 (1985).

[12] Barkhuijsen, H., De Beer, R., Bovée, W. M. M. J., Creyghton, J. H. N. & Van Ormondt, D. Application of linear prediction and singular value decomposition (LPSVD) to determine NMR frequencies and intensities from the FID. *Mag. Reson. Med.* **2**, 86–89 (1985).

[13] Barkhuijsen, H., de Beer, R. & van Ormondt, D. Improved algorithm for noniterative time-domain model fitting to exponentially damped magnetic resonance signals. *J. Mag. Reson.* **73**, 553 – 557 (1987).

[14] Hua, Y. & Sarkar, T. K. Matrix pencil method for estimating parameters of exponentially damped/undamped sinusoids in noise. *IEEE Trans. Acoust., Speech and Signal Process.* **38**, 814–824

(1990).

[15] Tang, J., Lin, C. P., Bowman, M. K. & Norris, J. R. An alternative to Fourier transform spectral analysis with improved resolution. *J. Mag. Reson.* **62**, 167 – 171 (1985).

[16] Hoch, J. C., Maciejewski, M. W., Mobli, M., Schuyler, A. D. & Stern, A. S. Maximum entropy reconstruction and nonuniform sampling in multidimensional NMR. *Acc. Chem. Res.* **47**, 708–717 (2014).

[17] Sibisi, S., Skilling, J., Brereton, R. G., Laue, E. D. & Staunton, J. Maximum entropy signal processing in practical NMR spectroscopy. *Nature (London)* **311**, 446–447 (1984).

[18] Lade, E. D., Skilling, J., Staunton, J., Sibisi, S. & Brereton, R. G. Maximum entropy method in nuclear magnetic resonance spectroscopy. *J. Mag. Reson.* **62**, 437 – 452 (1985).

[19] Bretthorst, G. L. Bayesian analysis. V. Amplitude estimation for multiple well-separated sinusoids. *J. Mag. Reson.* **98**, 501 – 523 (1992).

[20] Kotyk, J. J., Hoffman, N. G., Hutton, W. C., Bretthorst, G. L. & Ackerman, J. J. H. Comparison of Fourier and Bayesian analysis of NMR signals. I. Well-separated resonances (the single-frequency case). *J. Mag. Reson.* **98**, 483 – 500 (1992).

[21] Kotyk, J. J., Hoffman, N. G., Hutton, W. C., Bretthorst, G. L. & Ackerman, J. J. H. Comparison of fourier and bayesian analysis of NMR signals. II. Examination of truncated free induction decay NMR data. *J. Mag. Reson.* **116**, 1 – 9 (1995).

[22] Miller, M. I. & Greene, A. S. Maximum-likelihood estimation for nuclear magnetic resonance spectroscopy. *J. Mag. Reson.* **83**, 525 – 548 (1989).

[23] Umesh, S. & Tufts, D. W. Estimation of parameters of exponentially damped sinusoids using fast maximum likelihood estimation with application to NMR spectroscopy data. *IEEE Trans. Signal Process.* **44**, 2245–2259 (1996).

[24] Neuhauser, D. Bound state eigenfunctions from wave packets: Time$\rightarrow$ energy resolution. *J. Chem. Phys.* **93**, 2611–2616 (1990).

[25] Mandelshtam, V. A. FDM: The filter diagonalization method for data processing in NMR experiments. *Prog. Nucl. Mag. Reson. Spectrosc.* **38**, 159 – 196 (2001).

[26] Mandelshtam, V. A. & Taylor, H. S. Harmonic inversion of time signals and its applications. *J. Chem. Phys.* **107**, 6756–6769 (1997).

[27] Tellier, C., Guillou-Charpin, M., Le Botlan, D. & Pelissolo, F. Analysis of low-resolution, low-field NMR relaxation data with the Padé-Laplace method. *Mag. Reson. Chem.* **29**, 164–167 (1991).

[28] Halvorson, H. R. Padé-Laplace algorithm for sums of exponentials: Selecting appropriate exponential model and initial estimates for exponential fitting. In *Numerical Computer Methods*, vol. 210 of

Methods in Enzymology, 54 – 67 (Academic Press, 1992).

[29] Gesmar, H. & Led, J. J. Spectral estimation of complex time-domain NMR signals by linear prediction. *J. Mag. Reson.* **76**, 183 – 192 (1988).

[30] Stoica, P. & Moses, R. L. *Introduction to Spectral Analysis* (Upper Saddle River, NJ: Prentice Hall, 1997).

[31] Vetterli, M., Marziliano, P. & Blu, T. Sampling signals with finite rate of innovation. *IEEE Trans. Signal Process.* **50**, 1417–1428 (2002).

[32] Akaike, H. A new look at the statistical model identification. *IEEE Trans. Automatic Control* **19**, 716–723 (1974).

[33] Wax, M. & Kailath, T. Detection of signals by information theoretic criteria. *IEEE Trans. Acoustics, Speech, and Signal Process.* **33**, 387–392 (1985).

[34] Barron, A., Rissanen, J. & Yu, B. The minimum description length principle in coding and modeling. *IEEE Trans. Info. Theory* **44**, 2743–2760 (1998).

[35] Badeau, R., David, B. & Richard, G. A new perturbation analysis for signal enumeration in rotational invariance techniques. *IEEE Trans. Signal Process.* **54**, 450–458 (2006).

[36] Papy, J. M., Lathauwer, L. D. & Huffel, S. V. A shift invariance-based order-selection technique for exponential data modelling. *IEEE Signal Process. Lett.* **14**, 473–476 (2007).

[37] Bretthorst, G. L., Hutton, W. C., Garbow, J. R. & Ackerman, J. J. H. Exponential model selection (in NMR) using Bayesian probability theory. *Concepts in Mag. Reson. Part A* **27A**, 64–72 (2005).

[38] Blu, T., Dragotti, P. L., Vetterli, M., Marziliano, P. & Coulot, L. Sparse sampling of signal innovations. *IEEE Signal Process. Mag.* **25**, 31–40 (2008).

[39] Roy, R., Paulraj, A. & Kailath, T. ESPRIT– A subspace rotation approach to estimation of parameters of cisoids in noise. *IEEE Trans. Acoustics, Speech, and Signal Process.* **34**, 1340–1342 (1986).

[40] Paulraj, A., Roy, R. & Kailath, T. A subspace rotation approach to signal parameter estimation. *Proc. IEEE* **74**, 1044–1046 (1986).

[41] Roy, R. & Kailath, T. ESPRIT- Estimation of signal parameters via rotational invariance techniques. *IEEE Trans. Acoustics, Speech, and Signal Process.* **37**, 984–995 (1989).

[42] Eriksson, A., Stoica, P. & Soderstrom, T. Second-order properties of MUSIC and ESPRIT estimates of sinusoidal frequencies in high SNR scenarios. *IEE Proc. F - Radar and Signal Process.* **140**, 266–272 (1993).

[43] Williamson, M. P. Using chemical shift perturbation to characterize ligand binding. *Prog. Nucl. Mag. Reson. Spectrosc.* **73**, 1 – 16 (2013).
